# Supplementary material for: Fast automated detection of COVID-19 from medical images using convolutional neural networks
Source: Commun Biol. 2021 Jan 4;4:35. doi: 10.1038/s42003-020-01535-7 (PMC7782580; doi:10.1038/s42003-020-01535-7)
Supplement: Supplementary file 2 — Supplementary Information [file 42003_2020_1535_MOESM2_ESM.pdf]

Fast automated detection of COVID-19 from medical images using convolutional neural networks

Shuang Liang[1,], **Huixiang Liu**[1,], **Yu Gu**[2,3,4,*], **Xiuhua Guo**[5,6], **Hongjun Li**[7], **Li Li**[7], **Zhiyuan Wu**[5,6], **Mengyang Liu**[5,6], and **Lixin Tao**[5,6]

[1]School of Automation and Electrical Engineering, University of Science and Technology Beijing, Beijing 100083, China.
[2]Beijing Advanced Innovation Center for Soft Matter Science and Engineering, Beijing University of Chemical Technology, Beijing 100029, China.
[3]School of AutoMation, Guangdong University of Petrochemical Technology, Maoming 525000, Guangdong, China.
[4]Department of Chemistry, Institute of Inorganic and Analytical Chemistry, Goethe-University, 60438 Frankfurt, Germany.
[5]Department of Epidemiology and Health Statistics, School of Public Health, Capital Medical University, Beijing, China.
[6]Beijing Municipal Key Laboratory of Clinical Epidemiology, Capital Medical University, Beijing, China.
[7]Beijing Youan Hospital, Capital Medical University, Beijing, China.

[*]Corresponding author: Yu Gu:guyu@mail.buct.edu.cn

Supplementary information

Supplementary Note 1: Explaination of the expert groups

In China, medical education starts after high school and ranges from three to six years at the undergraduate level, followed by 3 years at the graduate level[1]. The 3-year postgraduate medical education is called standardized residency training (SRT) and is aimed at equiping medical graduates with practical clinical skills to enable them to become application-oriented, multi-skilled professionals serving in the national health system[2]. After passing the SRT, resident physicians can become a specialists. Students majoring in medical imaging discipline can enter the department of radiology as residents after 5-7 years of study at a college[3]. In our manuscript, the expert group is consisted of five members including a 7th-year respiratory resident, a 3rd-year emergency resident, a 1st-year respiratory intern, a 5th-year radiologist and a 3rd-year radiologist. Here, the 7th-year respiratory resident is a doctor that has passed the SRT and has 7 years of experience in the clinical work of respiratory diseases. The 3rd-year emergency resident is a doctor that has passed the SRT and has 3 years of experience in an emergency department. The 1st-year respiratory intern is a doctor that has passed the SRT and has started clinical work in respiratory diseases. The 5th-year radiologist has 5 years of experience in the department of radiology and the 3rd-year radiologist has 3 years of experience in the department of radiology.

Supplementary Note 2: Equations of the five metrics

The Kappa score (Kappa), sensitivity (Sen), specificity (Spe), precision (Pr), and F1-score metrics derived from the confusion matrix were used to determine the performance of the CNNCF. The equations are as follows:

$$pe = ((TN + FN) * (TN + FP) + (TP + FP) * (TP + FN))/(N * N) \tag{1}$$

$$p0 = (TP + TN)/N \tag{2}$$

$$Kappa = (p0 - pe)/(1 - pe) \tag{3}$$

$$Sen = TP/(TP + FN) \tag{4}$$

$$Spe = TN/(TN + FP) \tag{5}$$

$$Pr = (TP)/(TP + FP) \tag{6}$$

$$F1 - Score = 2 * Pr * Sen/(Pr + Sen) \tag{7}$$

where True positive (TP) represents the number of COVID-19 lung images correctly classified as COVID-19 cases and TN represents the number of *Normal lung images correctly classified as the *Normal lung cases. FP represents the number of *Normal lung images incorrectly classified as COVID-19 cases and FN represents the number of COVID-19 lung images misclassified as *Normal lung cases. N represents the number of cases in the test dataset.

Supplementary Figures

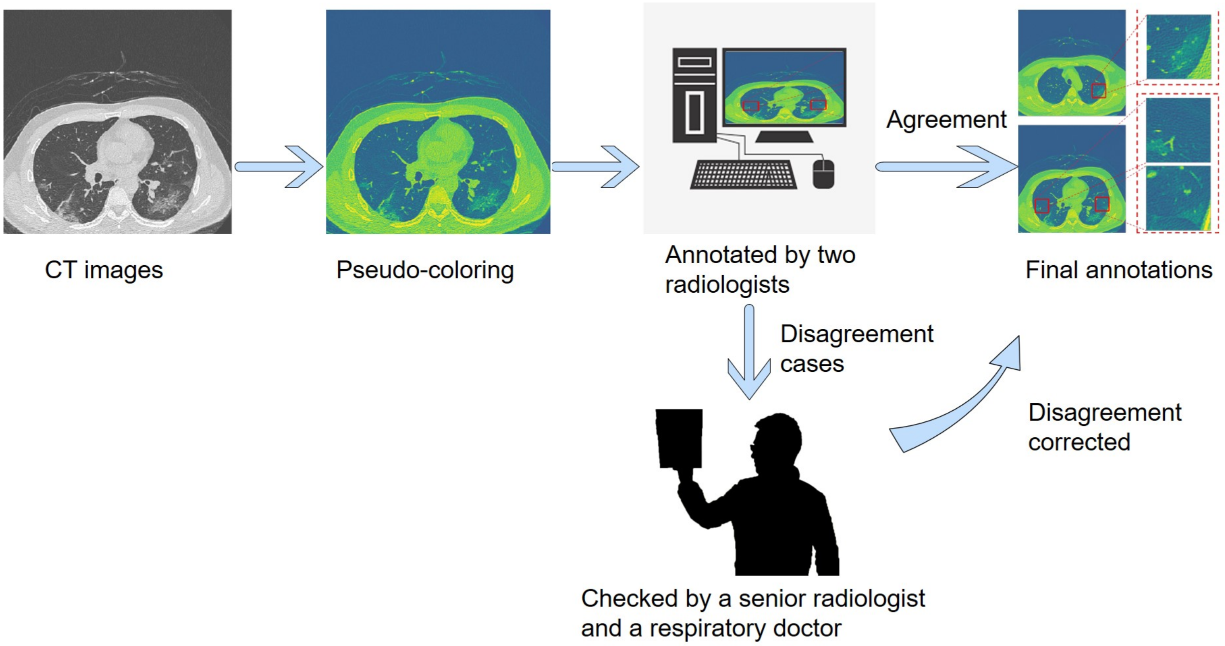

Supplementary Figure 1: The overall pipeline of the annotation

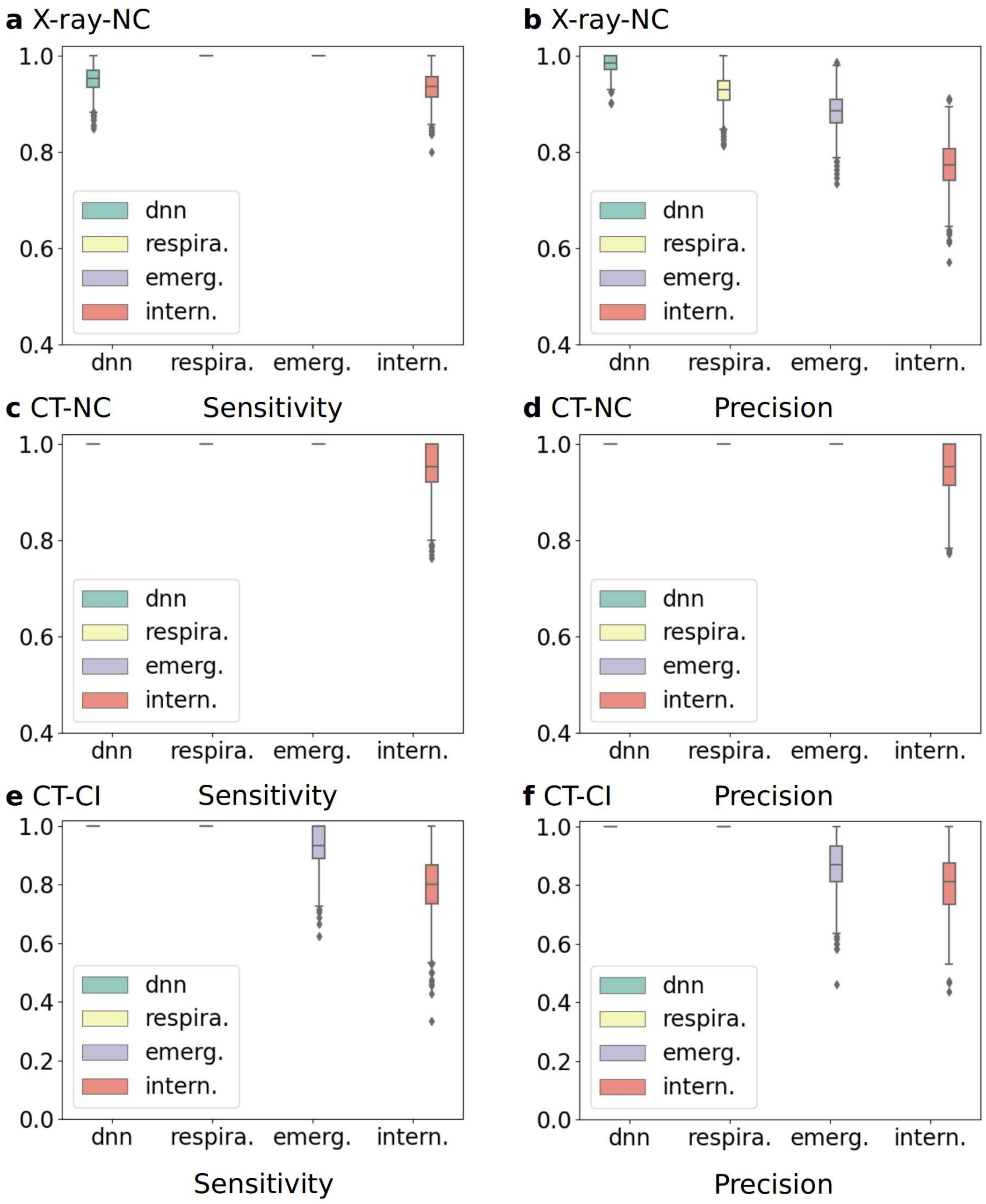

Supplementary Figure 2: Boxplots of precision and sensitivity for the CNNCF and expert results for COVID-19 identification. NC indicates that the positive case is a COVID-19 case, and the negative case is *Normal. CI indicates that the positive case is COVID-19, and the negative case is influenza. Bootstrapping is used to generate 1000 resampled validation sets for XPVS, CTPVS and CTHVS.

Supplementary Experiments and Tables

Supplementary Table 1: Results of McNemar's test for the CNNCF and expert results for COVID-19 and *Normal cases for the X-data collected from CCD and RSNA datasets

	CNNCF/Respira.		CNNCF/Emerg.		CNNCF/Intern.		CNNCF/Rad-5th.		CNNCF/Rad-3rd.	
	p-value	statistic	p-value	statistic	p-value	statistic	p-value	statistic	p-value	statistic
F1	1.0000	0.9725	1.0000	0.9323	1.0000	0.8421	1.0000	0.9667	1.0000	0.8308
Kappa	1.0000	0.8852	1.0000	0.9477	1.0000	0.6896	1.0000	0.9535	1.0000	0.7576
Specificity	1.0000	0.9625	1.0000	0.9371	1.0000	0.8859	1.0000	0.9934	1.0000	0.8774
Sensitivity	1.0000	0.9701	1.0000	0.9701	1.0000	0.9138	1.0000	0.9508	1.0000	0.9474
Precision	1.0000	0.9103	1.0000	0.8701	1.0000	0.8052	1.0000	0.9808	1.0000	0.7397

Supplementary Table 2: Results of McNemar's Test for CNNCF and experts on distinct of COVID-19 and *Normal cases by means of CT-data collected from Youan hospital and the LUNA-16 dataset

	CNNCF/Respira.		CNNCF/Emerg.		CNNCF/Intern.		CNNCF/Rad-5th.		CNNCF/Rad-3rd.	
	p-value	statistic	p-value	statistic	p-value	statistic	p-value	statistic	p-value	statistic
F1	1.0000	1.0000	1.0000	1.0000	1.0000	0.9710	1.0000	1.0000	1.0000	0.9333
Kappa	1.0000	1.0000	1.0000	1.0000	1.0000	0.9149	1.0000	1.0000	1.0000	0.8477
Specificity	1.0000	1.0000	1.0000	1.0000	1.0000	0.9630	1.0000	1.0000	1.0000	0.9412
Sensitivity	1.0000	1.0000	1.0000	1.0000	1.0000	0.9223	1.0000	1.0000	1.0000	0.9130
Precision	1.0000	1.0000	1.0000	1.0000	1.0000	0.9532	1.0000	1.0000	1.0000	0.9545

Supplementary Table 3: Results of McNemar's Test for CNNCF and experts on distinct of COVID-19 and influenza cases by means of CT-data collected from Youan hospital

	CNNCF/Respira.		CNNCF/Emerg.		CNNCF/Intern.		CNNCF/Rad-5th.		CNNCF/Rad-3rd.	
	p-value	statistic	p-value	statistic	p-value	statistic	p-value	statistic	p-value	statistic
F1	1.0000	1.0000	1.0000	0.8841	1.0000	0.8427	1.0000	0.9333	1.0000	0.8333
Kappa	1.0000	1.0000	1.0000	0.8551	1.0000	0.6260	1.0000	0.8837	1.0000	0.7473
Specificity	1.0000	1.0000	1.0000	0.9371	1.0000	0.8859	1.0000	0.9048	1.0000	0.9545
Sensitivity	1.0000	1.0000	1.0000	0.9506	1.0000	0.8022	1.0000	1.0000	1.0000	0.7692
Precision	1.0000	1.0000	1.0000	0.8541	1.0000	0.7327	1.0000	0.8750	1.0000	0.9091

a. Experiment-E. The results of the five evaluation indicators for the comparison of the COVID-19 cases and *Normal cases of the CTHVS are shown in Supplementary Table 1. The CNNCF exhibits good performance for the five evaluation indices, which are similar to that of the Respire., the Emerg. and the Rad-5th, and higher than that of the Intern and the Rad-3rd. The ROC scores are plotted in Supplementary Fig. 1-a; the AUROC of the CNNCF is 1.0. The precision-recall scores are shown in Supplementary Fig. 1-c; the AUPRC of the CNNCF is 1.0.

b. Experiment-F. The results of the five evaluation indicators for the comparison of the COVID-19 cases and pneumonia cases of the CTHVS are shown in Supplementary Table 1 where the *Normal cases are from CTPVS and the COVID-19 cases are from the CTHVS. The CNNCF exhibits good performance for the five evaluation indices, which are similar to that of the Respire. and higher than that of the Intern, the Emerg, the Rad-5th and the Rad-3rd. The ROC scores are plotted in Supplementary Fig. 1-b; the AUROC of the CNNCF is 1.0. The precision-recall scores are shown in Supplementary Fig. 1-d; the AUPRC of the CNNCF is 1.0.

c. Experiment-G. The boxplots of the five evaluation indicators, the F1 score, the kappa coefficient, and the specificity of experiment E-F are shown in Supplementary Fig. 2, and the precision and sensitivity are shown in the supplementary Supplementary Fig. 3. Bootstrapping method as introduced in the main manuscript was used to calculate the empirical distributions, and McNemar's test as introduced in the main manuscript was used to analyze the differences between the CNNCF and the experts. The p-values of the McNemar's test (Supplementary Table 2-3) for the five evaluation indicators were all 1.0.

Supplementary Table 4: Performance indices of the classification framework (CNNCF) of the experiments E-F and the average performance of the 7th year respiratory resident (Respira.), the 3rd year emergency resident (Emerg.), the 1st year respiratory intern (Intern), the 5th year radiologist(Rad-5th) and the 3rd year radiologist(Rad-3rd).

	CT(*Normal and COVID-19 cases from Youan hospital)					
	CNNCF	Respire.	Emerg.	Intern.	Rad-5th	Rad-3rd
F1(95%CI)	1.0000<br>(1.0000,1.0000)	1.0000<br>(1.0000,1.0000)	1.0000<br>(1.0000,1.0000)	0.9268<br>(0.8292,1.0000)	1.0000<br>(1.0000,1.0000)	0.9744<br>(0.9143,1.0000)
Kappa(95%CI)	1.0000<br>(1.0000,1.0000)	1.0000<br>(1.0000,1.0000)	1.0000<br>(1.0000,1.0000)	0.8500<br>(0.6700,1.0000)	1.0000<br>(1.0000,1.0000)	0.9500<br>(0.8429,1.0000)
Specificity(95%CI)	1.0000<br>(1.0000,1.0000)	1.0000<br>(1.0000,1.0000)	1.0000<br>(1.0000,1.0000)	0.9000<br>(0.7497,1.0000)	1.0000<br>(1.0000,1.0000)	1.0000<br>(1.0000,1.0000)
Sensitivity(95%CI)	1.0000<br>(1.0000,1.0000)	1.0000<br>(1.0000,1.0000)	1.0000<br>(1.0000,1.0000)	0.9500<br>(0.8333, 1.0000)	1.0000<br>(1.0000,1.0000)	0.9500<br>(0.8421,1.0000)
Precision(95%CI)	1.0000<br>(1.0000,1.0000)	1.0000<br>(1.0000,1.0000)	1.0000<br>(1.0000,1.0000)	0.9048<br>(0.7646,1.0000)	1.0000<br>(1.0000,1.0000)	1.0000<br>(1.0000,1.0000)
	CT(Pneumonia and COVID-19 cases from Youan hospital)					
	CNNCF	Respire.	Emerg.	Intern.	Rad-5th	Rad-3rd
F1(95%CI)	1.0000<br>(1.0000,1.0000)	1.0000<br>(1.0000,1.0000)	0.9048<br>(0.7907,0.9787)	0.8000<br>(0.6521,0.9143)	0.9744<br>(0.9129,1.0000)	0.7391<br>(0.5714,0.8627)
Kappa(95%CI)	1.0000<br>(1.0000,1.0000)	1.0000<br>(1.0000,1.0000)	0.8678<br>(0.7283,0.9703)	0.7158<br>(0.5357,0.8752)	0.9654<br>(0.8846,1.0000)	0.6266<br>(0.4398,0.8031)
Specificity(95%CI)	1.0000<br>(1.0000,1.0000)	1.0000<br>(1.0000,1.0000)	0.9455<br>(0.8823,1.0000)	0.8727<br>(0.7800,0.9592)	1.0000<br>(1.0000,1.0000)	0.8364<br>(0.7451,0.9299)
Sensitivity(95%CI)	1.0000<br>(1.0000,1.0000)	1.0000<br>(1.0000,1.0000)	0.9500<br>(0.8333,1.0000)	0.9000<br>(0.7598,1.0000)	0.9500<br>(0.8398,1.0000)	0.8500<br>(0.6842,1.0000)
Precision(95%CI)	1.0000<br>(1.0000,1.0000)	1.0000<br>(1.0000,1.0000)	0.8636<br>(0.7000,1.0000)	0.7200<br>(0.5357,0.8890)	1.0000<br>(1.0000,1.0000)	0.6538<br>(0.4686,0.8335)

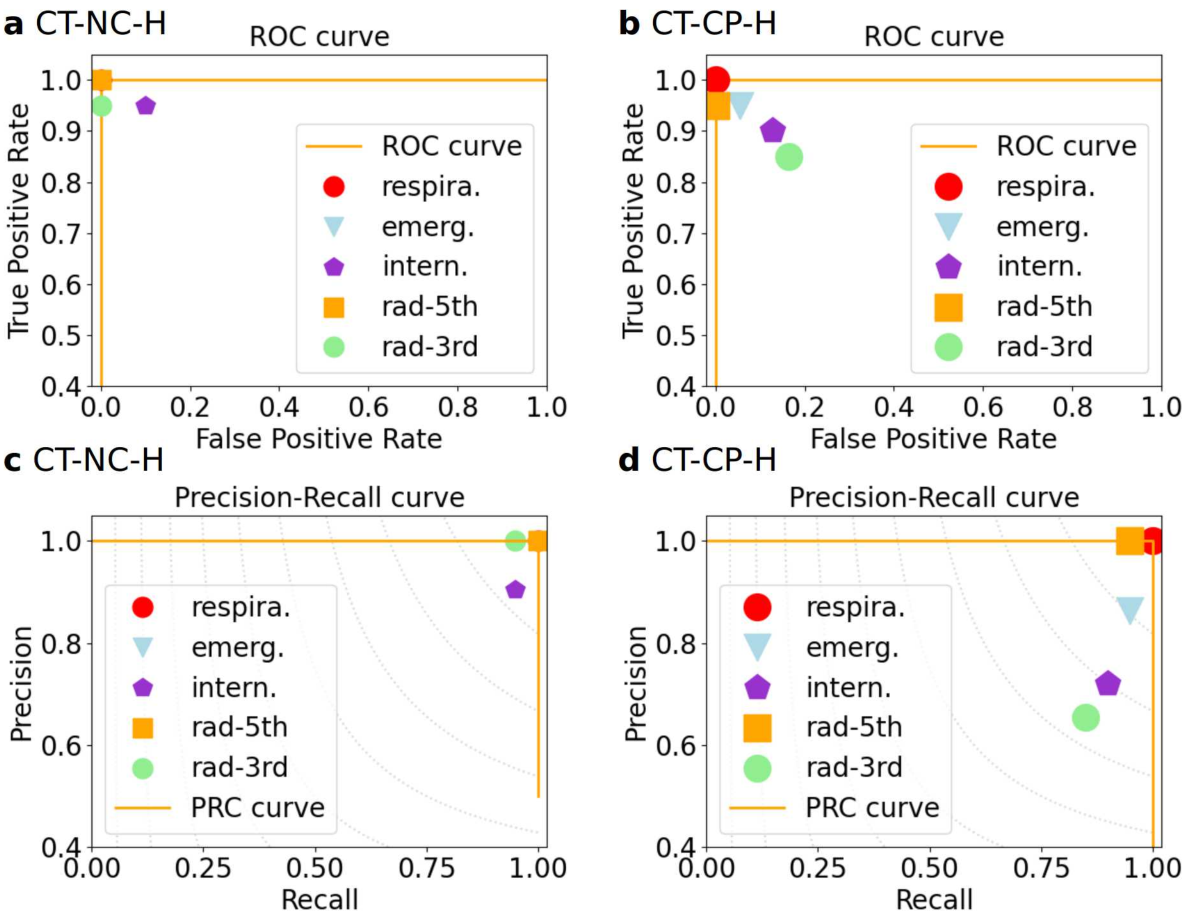

Supplementary Figure 3: ROC and PRC curves for the CNNCF and expert results for COVID-19 identification. NC indicates that the positive case is a COVID-19 case, and the negative case is *Normal. CP indicates that the positive case is COVID-19, and the negative case is pneumonia. H indicated that the cases are collected from Youan hospital. Bootstrapping is used to generate 1000 resampled validation sets for CTHVS.

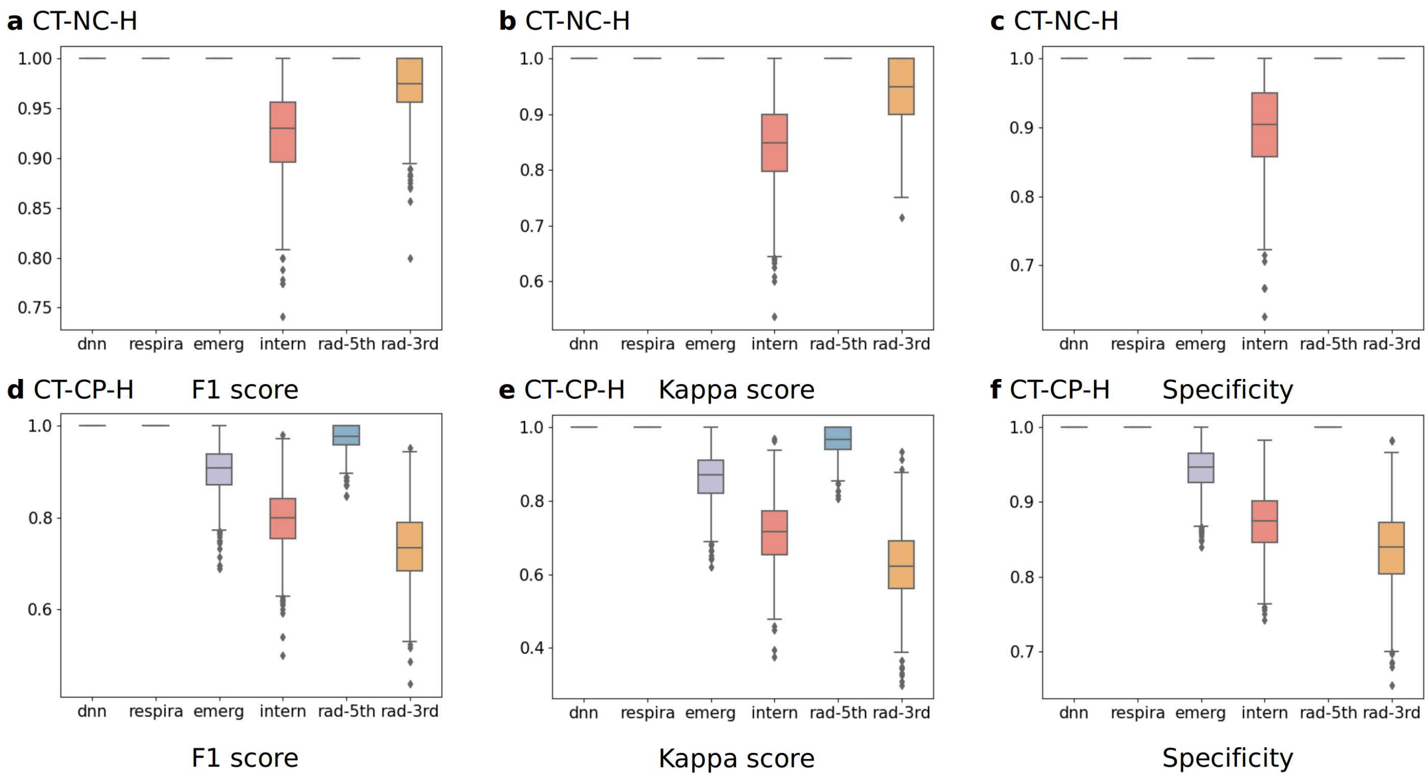

Supplementary Figure 4: Boxplots of f1-score, kappa score and specificity for the CNNCF and expert results for COVID-19 identification. NC indicates that the positive case is a COVID-19 case, and the negative case is *Normal. CP indicates that the positive case is COVID-19, and the negative case is pneumonia. H indicated that the cases are collected from Youan hospital. Bootstrapping is used to generate 1000 resampled validation sets for CTHVS.

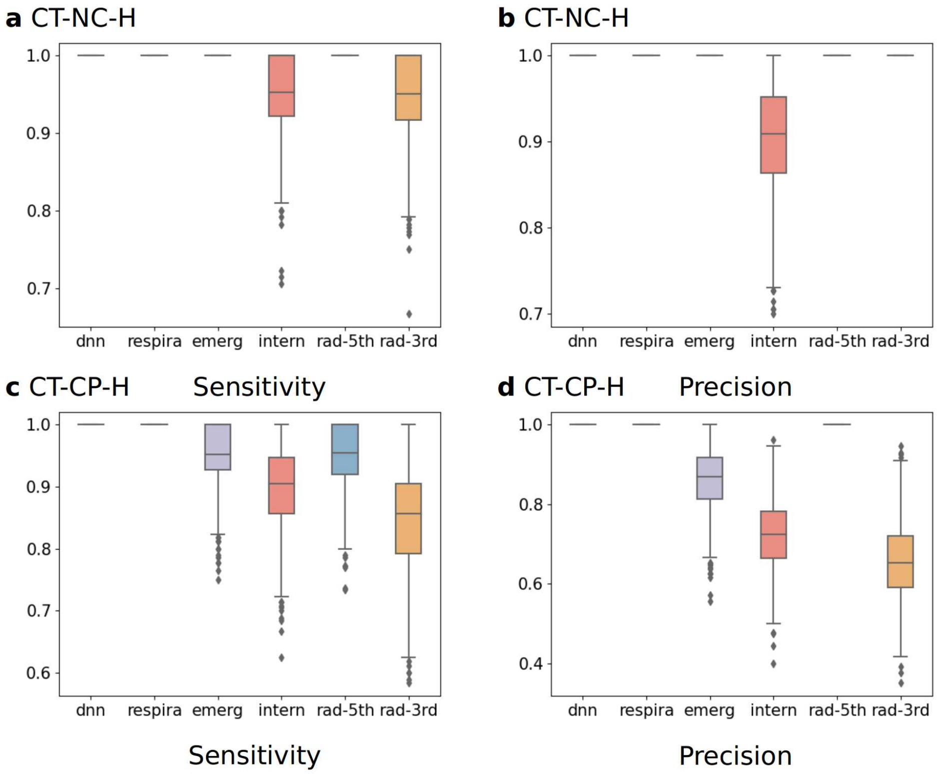

Supplementary Figure 5: Boxplots of precision and sensitivity for the CNNCF and expert results for COVID-19 identification. NC indicates that the positive case is a COVID-19 case, and the negative case is *Normal. CP indicates that the positive case is COVID-19, and the negative case is pneumonia. H indicated that the cases are collected from Youan hospital. Bootstrapping is used to generate 1000 resampled validation sets for CTHVS.

Supplementary Table 5: Results of McNemar's test for the CNNCF and expert results for COVID-19 and *Normal cases for the CT-data collected from Youan hospital

	CNNCF/Respira.		CNNCF/Emerg.		CNNCF/Intern.		CNNCF/Rad-5th.		CNNCF/Rad-3rd.	
	p-value	statistic	p-value	statistic	p-value	statistic	p-value	statistic	p-value	statistic
F1	1.0000	1.0000	1.0000	1.0000	1.0000	0.9200	1.0000	1.0000	1.0000	0.9778
Kappa	1.0000	1.0000	1.0000	1.0000	1.0000	0.7872	1.0000	1.0000	1.0000	0.9492
Specificity	1.0000	1.0000	1.0000	1.0000	1.0000	0.9286	1.0000	1.0000	1.0000	1.0000
Sensitivity	1.0000	1.0000	1.0000	1.0000	1.0000	0.8846	1.0000	1.0000	1.0000	0.9565
Precision	1.0000	1.0000	1.0000	1.0000	1.0000	0.9583	1.0000	1.0000	1.0000	1.0000

Supplementary Table 6: Results of McNemar's test for the CNNCF and expert results for COVID-19 and pneumonia cases for the CT-data collected from Youan hospital

	CNNCF/Respira.		CNNCF/Emerg.		CNNCF/Intern.		CNNCF/Rad-5th.		CNNCF/Rad-3rd.	
	p-value	statistic	p-value	statistic	p-value	statistic	p-value	statistic	p-value	statistic
F1	1.0000	1.0000	1.0000	0.9200	1.0000	0.7879	1.0000	1.0000	1.0000	0.8135
Kappa	1.0000	1.0000	1.0000	0.8801	1.0000	0.7298	1.0000	0.9683	1.0000	0.6954
Specificity	1.0000	1.0000	1.0000	0.9412	1.0000	0.9016	1.0000	1.0000	1.0000	0.8163
Sensitivity	1.0000	1.0000	1.0000	0.9583	1.0000	0.9286	1.0000	0.9565	1.0000	0.9231
Precision	1.0000	1.0000	1.0000	0.8846	1.0000	0.6842	1.0000	1.0000	1.0000	0.7273

d. Experiment-H. The results of the five evaluation indicators for the comparison of the COVID-19 cases and *Normal cases of the XHVS are shown in supplementery Table 4. The CNNCF exhibits good performance with the best score of specificity of 96.00% which was similar to that of the Respire.(96.00%) and the Rad-5th(96.00%), and higher than that of the Emerg.(88.00%), the Intern.(80.00%) and the Rad-3rd(84.00%). The F1 score was 90.00%, which was similar to that of the Rad-5th(90.00%), higher than that of the Emerg.(81.82%), the Intern (69.57%) and the Rad-3rd (72.73%), and lower than that of the Respire. (95.24%). The kappa score was 86.00%, which was similar to that of the Rad-5th(86.00%), higher than that of the Emerg.(73.58%), the Intern (55.05%) and the Rad-3rd (60.38%), and lower than that of the Respire (93.20%).The sensitivity index was 90.00%, which was similar to that of the Emerg.(90.00%) and the Rad-5th(90.00%), higher than that of the Intern (80.00%) and the Rad-3rd (80.00%), and lower than that of the Respire. (100%). The Precision index was 90.00%, which was similar to that of the Rad-5th(90.00%), higher than that of the Emerg.(75.00%), the Intern (61.54%) and the Rad-3rd (66.67%), and lower than that of the Respire. (90.91%). The ROC scores are plotted in Supplementary Fig. 4-a; the AUROC of the CNNCF is 0.9920. The precision-recall scores are shown in Supplementary Fig. 4-d; the AUPRC of the CNNCF is 0.9799.

e. Experiment-I. The results of the five evaluation indicators for the comparison of the COVID-19 cases and influenza cases of the XHVS are shown in Supplementary Table 4. The CNNCF exhibits good performance with the best score of specificity of 95.56%, and a precision of 81.82%. The F1 score was 85.71%, which was higher than that of the Rad-5th(81.82%), the Emerg.(80.00%), the Rad-3rd(64.00%) and the Intern.(59.26%) and lower than that of the Respire.(86.96%). The kappa score was 82.35%, which was higher than that of the Rad-5th(77.32%), the Emerg.(74.42%), the Rad-3rd(53.95%) and the Intern.(47.16%) and lower than that of the Respire.(83.58%). The sensitivity index was 90.00%, which was similar to that of the Rad-5th(90.00%), higher than that of the Rad-3rd(80.00%) and the Intern.(80.00%), and lower than that of the Respire.(100.00%) and the Emerg.(100.00%). The ROC scores are plotted in Supplementary Fig. 4-b; the AUROC of the CNNCF is 0.9956. The precision-recall scores are shown in Supplementary Fig. 4-e; the AUPRC of the CNNCF is 0.9799.

f. Experiment-J. The results of the five evaluation indicators for the comparison of the COVID-19 cases and pneumonia cases of the XHVS are shown in Supplementary Table 4. The CNNCF exhibits good

performance with the best score of specificity of 96.33%, and a precision of 81.82%. The F1 score was 85.71%, which was higher than that of the Emerg.(80.00%), the Rad-3rd(64.00%) and the Intern.(61.54%) and lower than that of the Respire.(86.96%) and the Rad-5th(86.96%). The kappa score was 82.97%, which was higher than that of the Emerg.(75.47%), the Rad-3rd(55.85%) and the Intern.(52.55%) and lower than that of the Respire.(84.21%) and the Rad-5th(84.21%). The sensitivity index was 90.00%, which was higher than that of the Rad-3rd(80.00%) and the Intern.(80.00%), and lower than that of the Respire.(100.00%), the Rad-5th(100.00%) and the Emerg.(100.00%). The ROC scores are plotted in Supplementary Fig. 4-c; the AUROC of the CNNCF is 0.9964. The precision-recall scores are shown in Supplementary Fig. 4-f; the AUPRC of the CNNCF is 0.9799.

g. Experiment-K. The boxplots of the five evaluation indicators, the F1 score, the kappa coefficient, and the specificity of experiment H-J are shown in Supplementary Fig. 5, and the precision and sensitivity are shown in the supplementary Supplementary Fig. 6. Bootstrapping method as introduced in the main manuscript was used to calculate the empirical distributions, and McNemar's test as introduced in the main manuscript was used to analyze the differences between the CNNCF and the experts. The p-values of the McNemar's test (Supplementary Table 5-7) for the five evaluation indicators were all 1.0.

Supplementary Table 7: Performance indices of the classification framework (CNNCF) of the experiments H-J and the average performance of the 7th year respiratory resident (Respira.), the 3rd year emergency resident (Emerg.), the 1st year respiratory intern (Intern), the 5th year radiologist(Rad-5th) and the 3rd year radiologist(Rad-3rd).

	X-data(*Normal and COVID-19 cases from Youan hospital)					
	CNNCF	Respire.	Emerg.	Intern.	Rad-5th	Rad-3rd
F1(95%CI)	0.9000 (0.7143,1.0000)	0.9524 (0.8182,1.0000)	0.8182 (0.5882,0.9600)	0.6957 (0.4286,0.8889)	0.9000 (0.7143,1.0000)	0.7273 (0.4346,0.9032)
Kappa(95%CI)	0.8600 (0.6181,1.0000)	0.9320 (0.7586,1.0000)	0.7358 (0.4615,0.9398)	0.5505 (0.2553,0.8248)	0.8600 (0.6390,1.0000)	0.6038 (0.6390,1.0000)
Specificity(95%CI)	0.9600 (0.8636,1.0000)	0.9600 (0.8750,1.0000)	0.8800 (0.7407,1.0000)	0.8000 (0.6400,0.9525)	0.9600 (0.8636,1.0000)	0.8400 (0.6667,0.9643)
Sensitivity(95%CI)	0.9000 (0.6667,1.0000)	1.0000 (1.0000,1.0000)	0.9000 (0.6667,1.0000)	0.8000 (0.5325,1.0000)	0.9000 (0.6667,1.0000)	0.8000 (0.5000,1.0000)
Precision(95%CI)	0.9000 (0.6667,1.0000)	0.9091 (0.6923,1.0000)	0.7500 (0.5000,1.0000)	0.6154 (0.3525,0.8750)	0.9000 (0.6917,1.0000)	0.6667 (0.3747,0.9231)
	X-data(Influenza and COVID-19 cases from Youan hospital)					
	CNNCF	Respire.	Emerg.	Intern.	Rad-5th	Rad-3rd
F1(95%CI)	0.8571 (0.6154,1.0000))	0.8696 (0.6667,1.0000)	0.8000 (0.5881,0.9524)	0.5926 (0.3222,0.8000)	0.8182 (0.6000,0.9600)	0.6400 (0.3529,0.8333)
Kappa(95%CI)	0.8235 (0.5611,1.0000)	0.8358 (0.6099,1.0000)	0.7442 (0.5244,0.9412)	0.4716 (0.1828,0.7176)	0.7732 (0.5154,0.9483)	0.5395 (0.2325,0.7732)
Specificity(95%CI)	0.9556 (0.8863,1.0000)	0.9333 (0.8478,1.0000)	0.8889 (0.7857,0.9773)	0.8000 (0.6665,0.9091)	0.9333 (0.8511,1.0000)	0.8444 (0.7380,0.9375)
Sensitivity(95%CI)	0.9000 (0.6667,1.0000)	1.0000 (1.0000,1.0000)	1.0000 (1.0000,1.0000)	0.8000 (0.5000,1.0000)	0.9000 (0.6667,1.0000)	0.8000 (0.5000,1.0000)
Precision(95%CI)	0.8182 (0.5333,1.0000)	0.7692 (0.5000,1.0000)	0.6667 (0.4167,0.9091)	0.4706 (0.2143,0.7143)	0.7500 (0.5000,1.0000)	0.5333 (0.2500,0.7827)
	X-data(Pneumonia and COVID-19 cases from Youan hospital)					
	CNNCF	Respire.	Emerg.	Intern.	Rad-5th	Rad-3rd
F1(95%CI)	0.8571 (0.6316,1.0000)	0.8696 (0.6956,1.0000)	0.8000 (0.5881,0.9524)	0.6154 (0.3199,0.8000)	0.8696 (0.6667,1.0000)	0.6400 (0.3636,0.8389)
Kappa(95%CI)	0.8297 (0.5761,1.0000)	0.8421 (0.6448,1.0000)	0.7547 (0.5301,0.9472)	0.5255 (0.2169,0.7405)	0.8421 (0.6242,1.0000)	0.5585 (0.2687,0.7979)
Specificity(95%CI)	0.9636 (0.9074,1.0000)	0.9455 (0.8800,1.0000)	0.9091 (0.8302,0.9815)	0.8545 (0.7500,0.9376)	0.9455 (0.8813,1.0000)	0.8727 (0.7736,0.9608)
Sensitivity(95%CI)	0.9000 (0.6667,1.0000)	1.0000 (1.0000,1.0000)	1.0000 (1.0000,1.0000)	0.8000 (0.5000,1.0000)	1.0000 (1.0000,1.0000)	0.8000 (0.5000,1.0000)
Precision(95%CI)	0.8182 (0.5556,1.0000)	0.7692 (0.5332,1.0000)	0.6667 (0.4165,0.9091)	0.5000 (0.2220,0.7333)	0.7692 (0.5000,1.0000)	0.5333 (0.2777,0.8000)

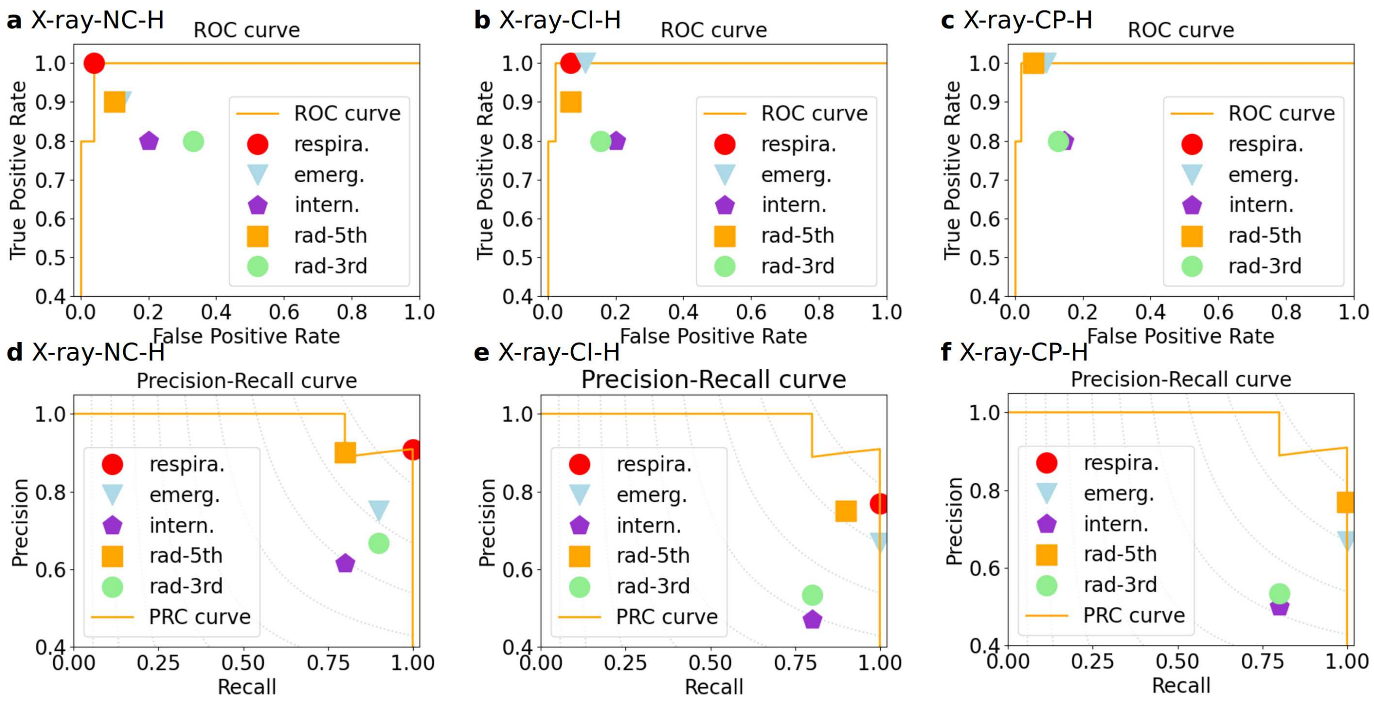

Supplementary Figure 6: ROC and PRC curves for the CNNCF and expert results for COVID-19 identification. NC indicates that the positive case is a COVID-19 case, and the negative case is *Normal. CI indicates that the positive case is COVID-19, and the negative case is influenza. CP indicates that the positive case is COVID-19, and the negative case is pneumonia. H indicated that the cases are collected from Youan hospital. Bootstrapping is used to generate 1000 resampled validation sets for XHVS.

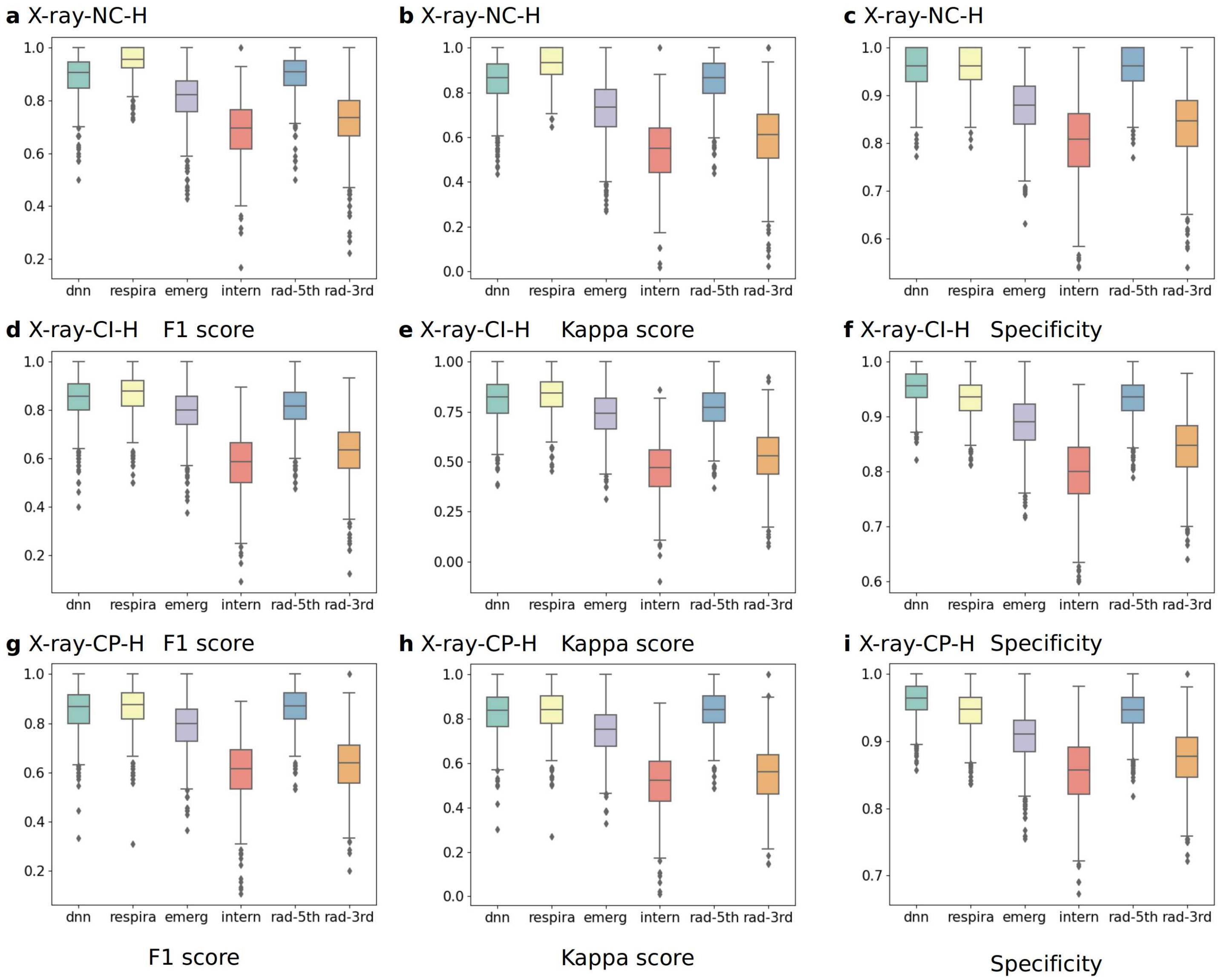

Supplementary Figure 7: Boxplots of f1-score, kappa score and specificity for the CNNCF and expert results for COVID-19 identification. NC indicates that the positive case is a COVID-19 case, and the negative case is *Normal. CI indicates that the positive case is COVID-19, and the negative case is influenza. CP indicates that the positive case is COVID-19, and the negative case is pneumonia. H indicated that the cases are collected from Youan hospital. Bootstrapping is used to generate 1000 resampled validation sets for XHVS.

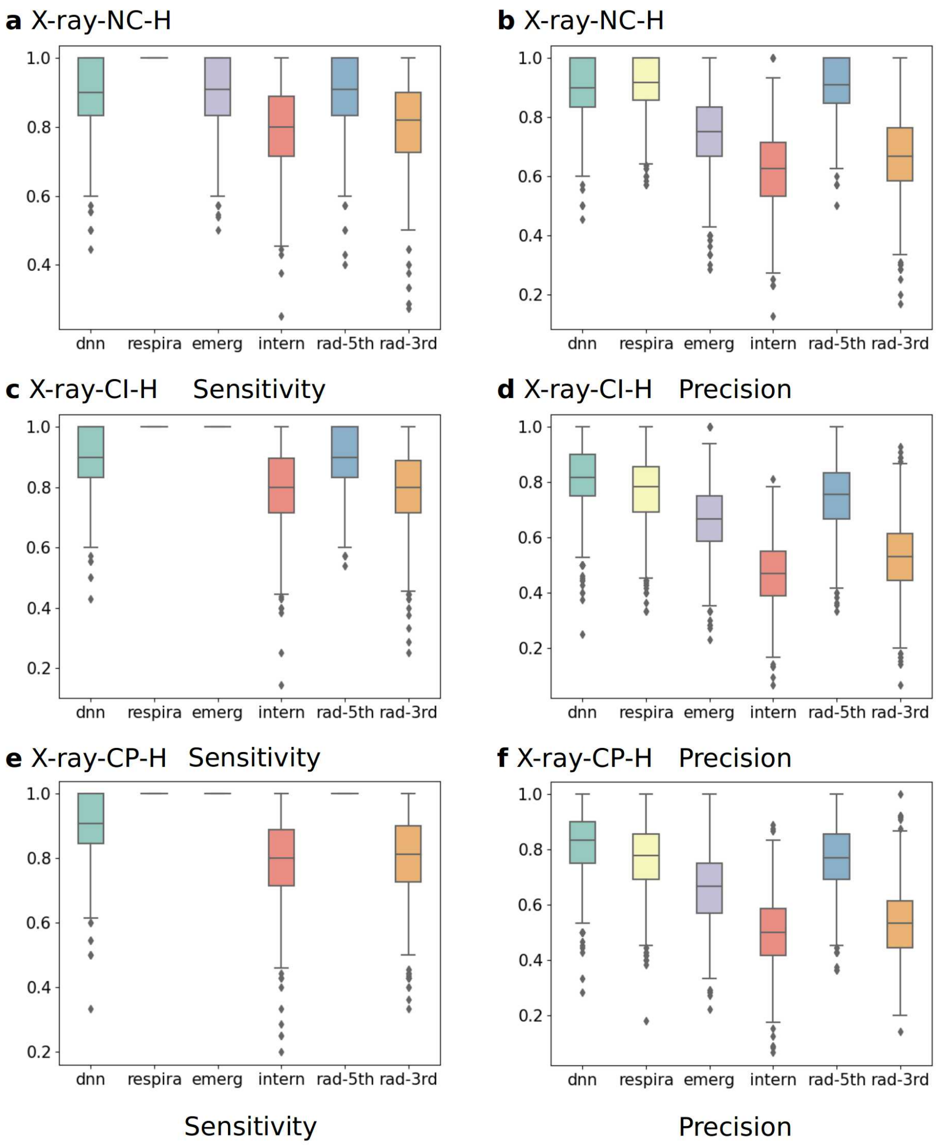

Supplementary Figure 8: Boxplots of precision and sensitivity for the CNNCF and expert results for COVID-19 identification. NC indicates that the positive case is a COVID-19 case, and the negative case is *Normal. CI indicates that the positive case is COVID-19, and the negative case is influenza. CP indicates that the positive case is COVID-19, and the negative case is pneumonia. H indicated that the cases are collected from Youan hospital. Bootstrapping is used to generate 1000 resampled validation sets for XHVS.

Supplementary Table 8: Results of McNemar's test for the CNNCF and expert results for COVID-19 and *Normal cases for the X-data collected from Youan hospital

	CNNCF/Respira.		CNNCF/Emerg.		CNNCF/Intern.		CNNCF/Rad-5th.		CNNCF/Rad-3rd.	
	p-value	statistic	p-value	statistic	p-value	statistic	p-value	statistic	p-value	statistic
F1	1.0000	0.8750	1.0000	0.7778	1.0000	0.8125	1.0000	0.8889	1.0000	0.8148
Kappa	1.0000	0.8387	1.0000	0.7059	1.0000	0.6557	1.0000	0.8511	1.0000	0.7009
Specificity	1.0000	0.9286	1.0000	0.8571	1.0000	0.8000	1.0000	1.0000	1.0000	0.8261
Sensitivity	1.0000	0.8333	1.0000	0.8333	1.0000	0.8333	1.0000	0.8333	1.0000	0.8333
Precision	1.0000	0.7778	1.0000	0.6364	1.0000	0.7647	1.0000	1.0000	1.0000	0.7333

Supplementary Table 9: Results of McNemar's test for the CNNCF and expert results for COVID-19 and influenza cases for the X-data collected from Youan hospital

	CNNCF/Respira.		CNNCF/Emerg.		CNNCF/Intern.		CNNCF/Rad-5th.		CNNCF/Rad-3rd.	
	p-value	statistic	p-value	statistic	p-value	statistic	p-value	statistic	p-value	statistic
F1	1.0000	0.8571	1.0000	0.7200	1.0000	0.5600	1.0000	0.7692	1.0000	0.7273
Kappa	1.0000	0.8243	1.0000	0.6458	1.0000	0.4434	1.0000	0.6984	1.0000	0.6598
Specificity	1.0000	0.9348	1.0000	0.8478	1.0000	0.8043	1.0000	0.9070	1.0000	0.9111
Sensitivity	1.0000	1.0000	1.0000	1.0000	1.0000	0.7778	1.0000	0.8333	1.0000	0.8000
Precision	1.0000	0.7500	1.0000	0.5625	1.0000	0.4375	1.0000	0.7143	1.0000	0.6667

Supplementary Table 10: Results of McNemar's test for the CNNCF and expert results for COVID-19 and pneumonia cases for the X-data collected from Youan hospital

	CNNCF/Respira.		CNNCF/Emerg.		CNNCF/Intern.		CNNCF/Rad-5th.		CNNCF/Rad-3rd.	
	p-value	statistic	p-value	statistic	p-value	statistic	p-value	statistic	p-value	statistic
F1	1.0000	0.8889	1.0000	0.7778	1.0000	0.7143	1.0000	0.8333	1.0000	0.5926
Kappa	1.0000	0.8713	1.0000	0.7441	1.0000	0.6404	1.0000	0.7969	1.0000	0.4874
Specificity	1.0000	0.9636	1.0000	0.9310	1.0000	0.8704	1.0000	0.9273	1.0000	0.8679
Sensitivity	1.0000	1.0000	1.0000	1.0000	1.0000	0.9091	1.0000	1.0000	1.0000	0.6667
Precision	1.0000	0.8000	1.0000	0.6364	1.0000	0.5882	1.0000	0.7143	1.0000	0.5333

h. Experiment-L. The results of the five evaluation indicators for the comparison of the pneumonia cases and the *Normal cases of the XPVS are shown in Supplementary Table 8. The CNNCF exhibits good performance with the best score of F1 score of 97.49%, a kappa score of 95.00%, a specificity of 98.00% and a precision of 97.98%. The sensitivity index was 97.00%, which was similar to that of the Rad-5th(97.00%), higher than that of the Emerg.(96.04%), Rad-3rd(94.00%) and the Intern.(93.00%), and lower than that of the Respire.(98.00%). The ROC scores are plotted in Supplementary Fig. 7-a; the AUROC of the CNNCF is 0.9970. The precision-recall scores are shown in Supplementary Fig. 7-c; the AUPRC of the CNNCF is 0.9964.

i. Experiment-M. The results of the five evaluation indicators for the comparison of the *Normal cases and the pneumonia cases of the CTPVS are shown in Supplementary Table 8. The CNNCF exhibits good performance for the five evaluation indices, which are similar to that of the Respire., the Emerg. and the Rad-5th and higher than that of the Intern and the Rad-3rd. The ROC scores are plotted in Supplementary Fig. 7-b; the AUROC of the CNNCF is 1.0. The precision-recall scores are shown in Supplementary Fig. 7-d; the AUPRC of the CNNCF is 1.0.

j. Experiment-N. The boxplots of the five evaluation indicators, the F1 score, the kappa coefficient, and the specificity of experiment L-M are shown in supplementary Fig. 8, and the precision and sensitivity are shown in the supplementary Fig. 9. Bootstrapping method as introduced in the main manuscript was used to calculate the empirical distributions, and McNemar's test as introduced in the main manuscript was used to analyze the differences between the CNNCF and the experts. The p-values of the McNemar's test (Supplementary Table 9-10) for the five evaluation indicators were all 1.0.

Supplementary Table 11: Performance indices of the classification framework (CNNCF) of the experiments L-M and the average performance of the 7th year respiratory resident (Respira.), the 3rd year emergency resident (Emerg.), the 1st year respiratory intern (Intern), the 5th year radiologist(Rad-5th) and the 3rd year radiologist(Rad-3rd).

	X-data(Pneumonia and *Normal cases from RSNA dataset)					
	CNNCF	Respire.	Emerg.	Intern.	Rad-5th	Rad-3rd
F1(95%CI)	0.9749<br>(0.9508,0.9951)	0.9703<br>(0.9442,0.9905)	0.9510<br>(0.9159,0.9792)	0.9163<br>(0.8764,0.9540)	0.9700<br>(0.9456,0.9901)	0.9307<br>(0.8950,0.9622)
Kappa(95%CI)	0.9500<br>(0.8999,0.9899)	0.9400<br>(0.8896,0.9800)	0.9091<br>(0.8387,0.9595)	0.8300<br>(0.7500,0.9004)	0.9400<br>(0.8900,0.9800)	0.8600<br>(0.7899,0.9200)
Specificity(95%CI)	0.9800<br>(0.9490,1.0000)	0.9600<br>(0.9174,0.9909)	0.9400<br>(0.8900,0.9810)	0.9000<br>(0.8381,0.9550)	0.9700<br>(0.9346,1.0000)	0.9200<br>(0.8627,0.9700)
Sensitivity(95%CI)	0.9700<br>(0.9327,1.0000)	0.9800<br>(0.9490,1.0000)	0.9604<br>(0.9175,0.9904)	0.9300<br>(0.8735,0.9727)	0.9700<br>(0.9314,1.0000)	0.9400<br>(0.8925,0.9806)
Precision(95%CI)	0.9798<br>(0.9478,1.0000)	0.9608<br>(0.9216,0.9907)	0.9417<br>(0.8952,0.9815)	0.9029<br>(0.8400,0.9529)	0.9700<br>(0.9340,1.0000)	0.9216<br>(0.8667,0.9688)
	CT(Pneumonia and *Normal cases from ICPNP and LUNA-16)					
	CNNCF	Respire.	Emerg.	Intern.	Rad-5th	Rad-3rd
F1(95%CI)	1.0000<br>(1.0000,1.0000)	1.0000<br>(1.0000,1.0000)	1.0000<br>(1.0000,1.0000)	0.9268<br>(0.8204,1.0000)	1.0000<br>(1.0000,1.0000)	0.9756<br>(0.9143,1.0000)
Kappa(95%CI)	1.0000<br>(1.0000,1.0000)	1.0000<br>(1.0000,1.0000)	1.0000<br>(1.0000,1.0000)	0.8500<br>(0.6500,1.0000)	1.0000<br>(1.0000,1.0000)	0.9500<br>(0.8387,1.0000)
Specificity(95%CI)	1.0000<br>(1.0000,1.0000)	1.0000<br>(1.0000,1.0000)	1.0000<br>(1.0000,1.0000)	0.9000<br>(0.7500,1.0000)	1.0000<br>(1.0000,1.0000)	0.9500<br>(0.8333,1.0000)
Sensitivity(95%CI)	1.0000<br>(1.0000,1.0000)	1.0000<br>(1.0000,1.0000)	1.0000<br>(1.0000,1.0000)	0.9500<br>(0.8333,1.0000)	1.0000<br>(1.0000,1.0000)	1.0000<br>(1.0000,1.0000)
Precision(95%CI)	1.0000<br>(1.0000,1.0000)	1.0000<br>(1.0000,1.0000)	1.0000<br>(1.0000,1.0000)	0.9048<br>(0.7725,1.0000)	1.0000<br>(1.0000,1.0000)	0.9524<br>(0.8421,1.0000)

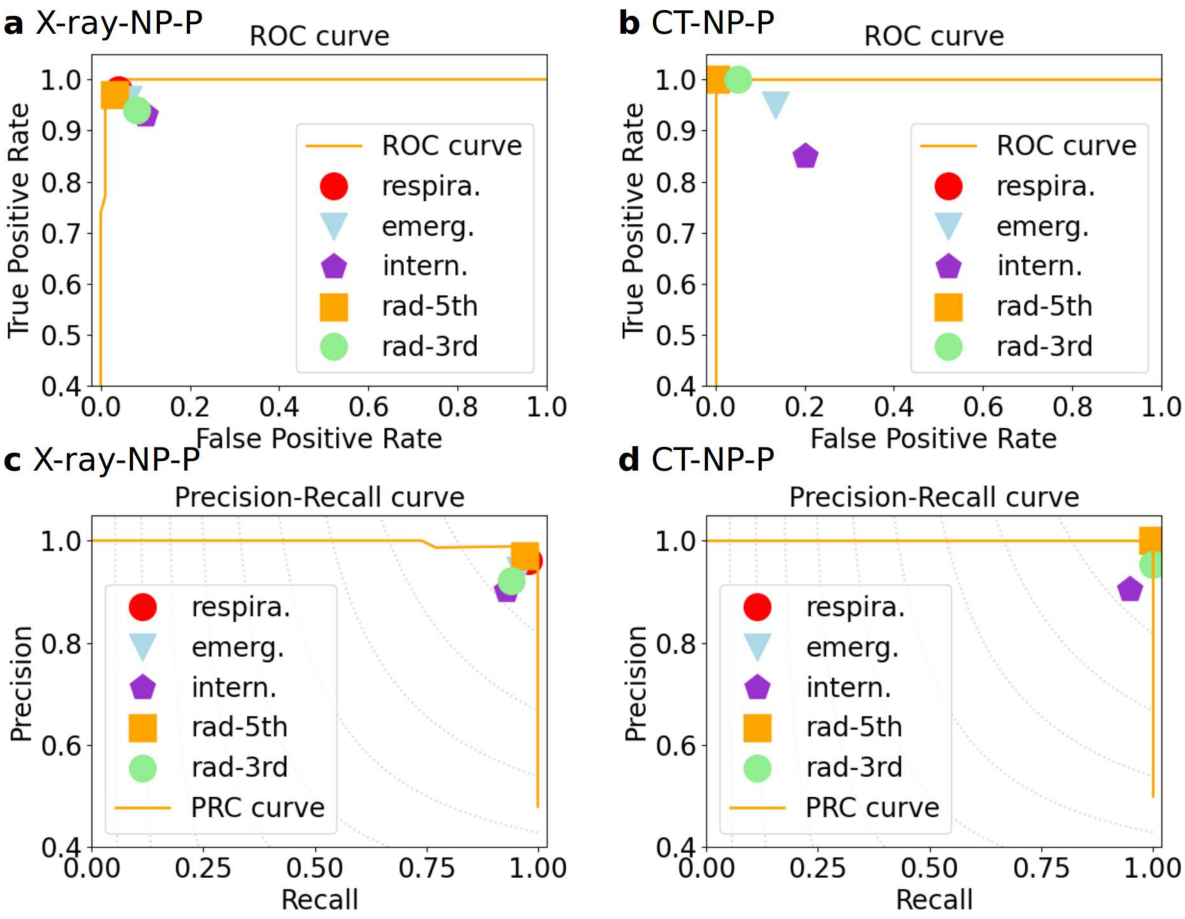

Supplementary Figure 9: ROC and PRC curves for the CNNCF and expert results for COVID-19 identification. NP indicates that the positive case is a pneumonia case, and the negative case is *Normal. CP indicates that the positive case is COVID-19, and the negative case is pneumonia. P indicated that the cases are collected from public datasets. Bootstrapping is used to generate 1000 resampled validation sets for XPVS and CTPVS.

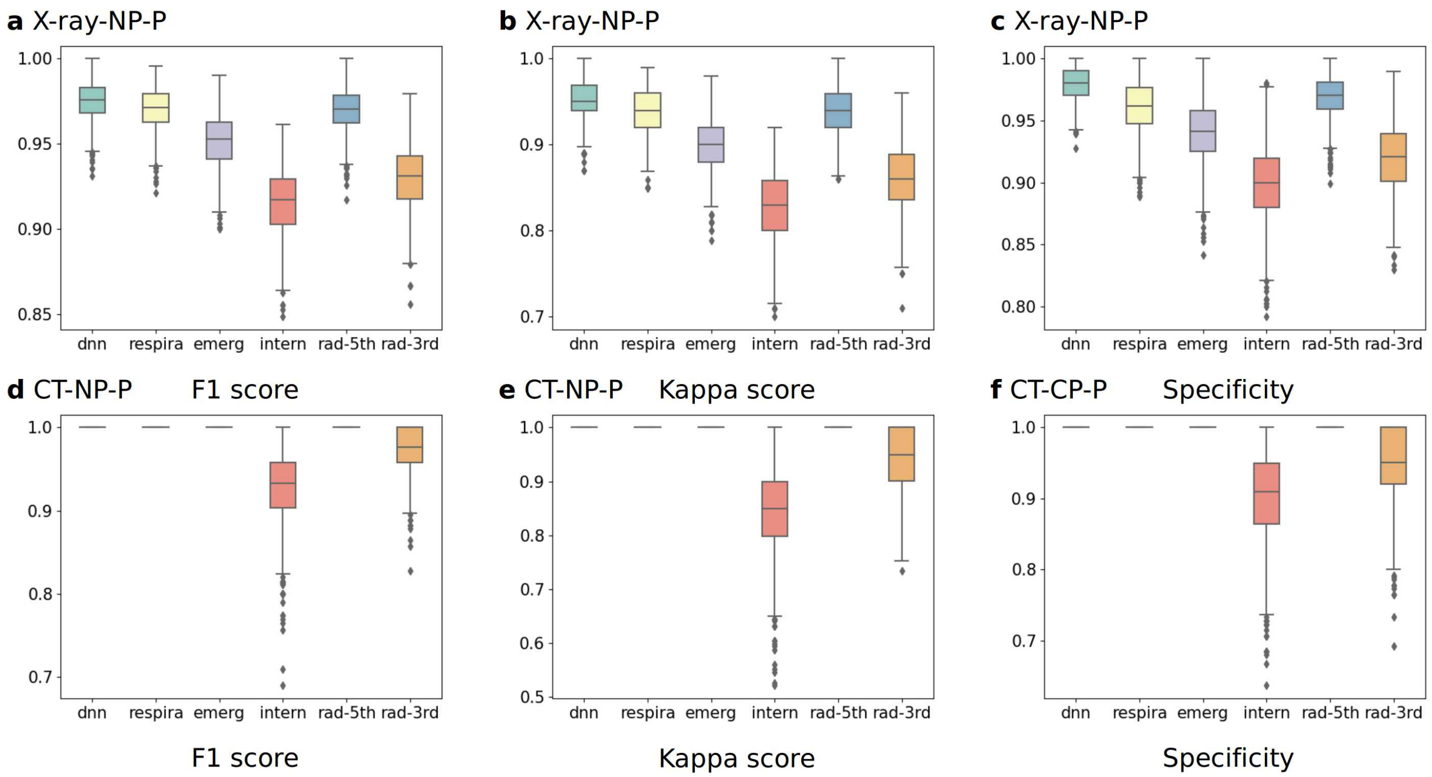

Supplementary Figure 10: Boxplots of f1-score, kappa score and specificity for the CNNCF and expert results for pneumonia identification. NP indicates that the positive case is a pneumonia case, and the negative case is *Normal. P indicated that the cases are collected from public datasets. Bootstrapping is used to generate 1000 resampled validation sets for XPVS and CTPVS.

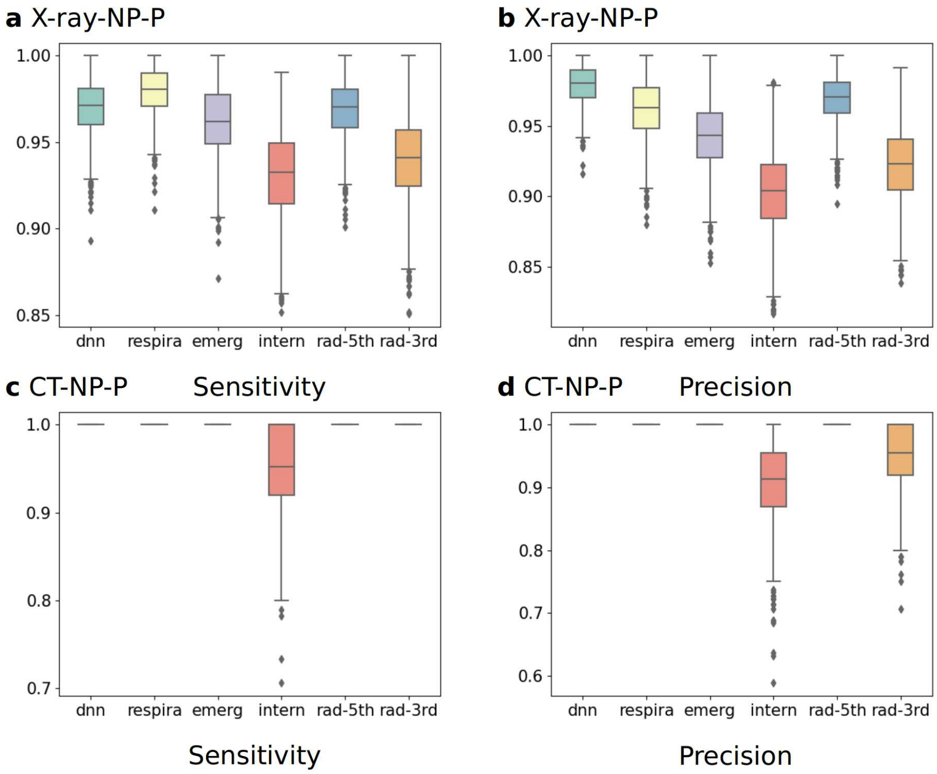

Supplementary Figure 11: Boxplots of precision and sensitivity for the CNNCF and expert results for pneumonia identification. NP indicates that the positive case is a pneumonia case, and the negative case is *Normal. P indicated that the cases are collected from public datasets. Bootstrapping is used to generate 1000 resampled validation sets for XPVS and CTPVS.

Supplementary Table 12: Results of McNemar's test for the CNNCF and expert results for pneumonia and *Normal cases for the X-data collected from RSNA dataset

	CNNCF/Respira.		CNNCF/Emerg.		CNNCF/Intern.		CNNCF/Rad-5th.		CNNCF/Rad-3rd.	
	p-value	statistic	p-value	statistic	p-value	statistic	p-value	statistic	p-value	statistic
F1	1.0000	0.9770	1.0000	0.9359	1.0000	0.8854	1.0000	0.9738	1.0000	0.9458
Kappa	1.0000	0.9593	1.0000	0.8701	1.0000	0.7798	1.0000	0.9499	1.0000	0.8901
Specificity	1.0000	0.9911	1.0000	0.9109	1.0000	0.9208	1.0000	0.9808	1.0000	0.9029
Sensitivity	1.0000	0.9659	1.0000	0.9596	1.0000	0.8586	1.0000	0.9659	1.0000	0.9659
Precision	1.0000	0.9884	1.0000	0.9135	1.0000	0.9140	1.0000	0.9789	1.0000	0.9057

Supplementary Table 13: Results of McNemar's test for the CNNCF and expert results for pneumonia and *Normal cases for the CT-data collected from ICNP and LUNA-16 dataset

	CNNCF/Respira.		CNNCF/Emerg.		CNNCF/Intern.		CNNCF/Rad-5th.		CNNCF/Rad-3rd.	
	p-value	statistic	p-value	statistic	p-value	statistic	p-value	statistic	p-value	statistic
F1	1.0000	1.0000	1.0000	1.0000	1.0000	0.8947	1.0000	1.0000	1.0000	0.9756
Kappa	1.0000	1.0000	1.0000	1.0000	1.0000	0.8000	1.0000	1.0000	1.0000	0.9500
Specificity	1.0000	1.0000	1.0000	1.0000	1.0000	0.8636	1.0000	1.0000	1.0000	0.9500
Sensitivity	1.0000	1.0000	1.0000	1.0000	1.0000	0.9444	1.0000	1.0000	1.0000	1.0000
Precision	1.0000	1.0000	1.0000	1.0000	1.0000	0.8500	1.0000	1.0000	1.0000	1.0000

k. Experiment-O. The results of the five evaluation indicators for the comparison of the pneumonia cases, the *Normal cases and the COVID-19 cases of the XMVS are shown in Supplementary Table 11. The CNNCF exhibits good performance on distinct of *Normal and COVID-19 cases with the best score of specificity of 98.86% and a precision of 97.14%. The F1 score was 95.77%, which was similar to that of the Respire.(96.00%), higher than that of the Emerg.(92.21%), Rad-3rd(84.08%) and the Intern.(82.50%), and lower than that of the Rad-5th(97.26%). The kappa score was 94.07%, which was similar to that of the Respire.(94.26%), higher than that of the Emerg.(88.70%), Rad-3rd(76.73%) and the Intern.(74.24%), and lower than that of the Rad-5th(96.11%). The specificity of 98.00% The sensitivity index was 94.44%, which was higher than that of Rad-3rd(91.67%) and the Intern.(91.67%), and lower than that of the Rad-5th(98.61%),the Emerg.(98.61%), and the Respire.(100.00%). Similar performance of the CNNCF was aslo achieved on distinct of Pneumonia and COVID-19 cases which was also shown in Table 11. The ROC scores for distinguishing COVID-19 from *Normal cases are plotted in Supplementary Fig. 10-a; the AUROC of the CNNCF is 0.9972. The precision-recall scores for distinguishing COVID-19 from *Normal cases are shown in Supplementary Fig. 10-c; the AUPRC of the CNNCF is 0.9948. The ROC scores for distinguishing COVID-19 from Pneumonia cases are plotted in Supplementary Fig. 10-b; the AUROC of the CNNCF is 0.9943. The precision-recall scores for distinguishing COVID-19 from Pneumonia cases are shown in Supplementary Fig. 10-d; the AUPRC of the CNNCF is 0.9899.

l. Experiment-P. The results of the five evaluation indicators for the comparison of the pneumonia cases, the *Normal cases and the COVID-19 cases of the CTMVS are shown in Supplementary Table 14. The CNNCF exhibits good performance on distinct of *Normal and COVID-19 cases for the five evaluation indices, which are similar to that of the Respire., the Emerg. and the Rad-5th and higher than that of the Intern and the Rad-3rd. Similar performance of the CNNCF was aslo achieved on distinct of Pneumonia and COVID-19 cases which was also shown in Table 14. The ROC scores for distinguishing COVID-19 from *Normal cases are plotted in Supplementary Fig. 11-a; the AUROC of the CNNCF is 1.0. The precision-recall scores for distinguishing COVID-19 from *Normal cases are shown in Supplementary Fig. 11-c; the AUPRC of the CNNCF is 1.0. The ROC scores for distinguishing COVID-19 from Pneumonia cases are plotted in Supplementary Fig. 11-b; the AUROC of the CNNCF is 0.9991. The precision-recall scores for distinguishing COVID-19 from Pneumonia cases are shown in Supplementary Fig. 11-d; the

AUPRC of the CNNCF is 0.9997.

m. Experiment-Q. The boxplots of the five evaluation indicators, the F1 score, the kappa coefficient, and the specificity of experiment O-P are shown in supplementary Fig. 12 and Fig. 13, and the precision and sensitivity are shown in the supplementary Fig. 14 and Fig. 15. Bootstrapping method as introduced in the main manuscript was used to calculate the empirical distributions, and McNemar's test as introduced in the main manuscript was used to analyze the differences between the CNNCF and the experts. The p-values of the McNemar's test (Supplementary Table 12,13,15 and 16) for the five evaluation indicators were all 1.0.

Supplementary Table 14: Performance indices of the classification framework (CNNCF) of the experiment O and the average performance of the 7th year respiratory resident (Respira.), the 3rd year emergency resident (Emerg.), the 1st year respiratory intern (Intern), the 5th year radiologist(Rad-5th) and the 3rd year radiologist(Rad-3rd).

	X-data(*Normal and COVID-19 cases from RSNA, CCD and Youan Hospital)					
	CNNCF	Respire.	Emerg.	Intern.	Rad-5th	Rad-3rd
F1(95%CI)	0.9577	0.9600	0.9221	0.8250	0.9726	0.8408
	(0.9189,0.9857)	(0.9206,0.9878)	(0.8740,0.9618)	(0.7612,0.8861)	(0.9427,0.9934))	(0.7702,0.9000)
Kappa(95%CI)	0.9407	0.9426	0.8870	0.7424	0.9611	0.7673
	(0.8851,0.9801)	(0.8871,0.9817)	(0.8199,0.9434)	(0.6573,0.8302)	(0.9188,0.9905)	(0.6730,0.8513)
Specificity(95%CI)	0.9886	0.9657	0.9371	0.8743	0.9829	0.8914
	(0.9714,1.0000)	(0.9349,0.9884)	(0.9000,0.9718)	(0.8239,0.9226)	(0.9605,1.0000)	(0.8424,0.9368)
Sensitivity(95%CI)	0.9444	1.0000	0.9861	0.9167	0.9861	0.9167
	(0.8857,0.9877)	(1.0000,1.0000)	(0.9529,1.0000)	(0.8511,0.9726)	(0.9487,1.0000)	(0.8450,0.9769)
Precision(95%CI)	0.9714	0.9231	0.8659	0.7500	0.9595	0.7765
	(0.9259,1.0000)	(0.8529,0.9759)	(0.7867,0.9342)	(0.6667,0.8427)	(0.9103,1.0000)	(0.6818,0.8605)
	X-data(Pneumonia and COVID-19 cases from RSNA and Youan Hospital)					
	CNNCF	Respire.	Emerg.	Intern.	Rad-5th	Rad-3rd
F1(95%CI)	0.9636	0.9600	0.9345	0.8821	0.9596	0.8987
	(0.9368,0.9862)	(0.9314,0.9831)	(0.8981,0.9655)	(0.8354,0.9223)	(0.9302,0.9846)	(0.8550,0.9372)
Kappa(95%CI)	0.9378	0.9305	0.8915	0.7926	0.9303	0.8229
	(0.8927,0.9766)	(0.8831,0.9694)	(0.8252,0.9387)	(0.7140,0.8633)	(0.8828,0.9757)	(0.7500,0.8864)
Specificity(95%CI)	0.9742	0.9548	0.9290	0.8839	0.9613	0.9032
	(0.9480,0.9940)	(0.9195,0.9857)	(0.8854,0.9660)	(0.8333,0.9299)	(0.9308,0.9929)	(0.8581,0.9497)
Sensitivity(95%CI)	0.9636	0.9818	0.9640	0.9182	0.9727	0.9273
	(0.9262,0.9913)	(0.9524,1.0000)	(0.9259,0.9915)	(0.8627,0.9646)	(0.9380,1.0000)	(0.8738,0.9712)
Precision(95%CI)	0.9636	0.9391	0.9068	0.8487	0.9469	0.8718
	(0.9266,0.9917)	(0.8916,0.9802)	(0.8509,0.9565)	(0.7788,0.9068)	(0.9038,0.9904)	(0.8087,0.9280)

Supplementary Table 15: Results of McNemar's Test for CNNCF and experts on distinct of COVID-19 and *Normal cases by means of X-data collected from RSNA, CCD datasets and Youan Hospital

	CNNCF/Respira.		CNNCF/Emerg.		CNNCF/Intern.		CNNCF/Rad-5th.		CNNCF/Rad-3rd.	
	p-value	statistic	p-value	statistic	p-value	statistic	p-value	statistic	p-value	statistic
F1	1.0000	0.9375	1.0000	0.9156	1.0000	0.9770	1.0000	0.9231	1.0000	0.9481
Kappa	1.0000	0.9259	1.0000	0.8901	1.0000	0.9302	1.0000	0.9231	1.0000	0.8621
Specificity	1.0000	0.7943	1.0000	0.7152	1.0000	0.8617	1.0000	0.9231	1.0000	0.6829
Sensitivity	1.0000	0.9375	1.0000	0.9148	1.0000	0.9708	1.0000	0.9231	1.0000	0.9351
Precision	1.0000	0.8800	1.0000	0.8282	1.0000	0.9157	1.0000	0.9231	1.0000	0.8148

Supplementary Table 16: Results of McNemar's Test for CNNCF and experts on distinct of COVID-19 and Pneumonia cases by means of X-data collected from RSNA dataset and Youan Hospital)

	CNNCF/Respira.		CNNCF/Emerg.		CNNCF/Intern.		CNNCF/Rad-5th.		CNNCF/Rad-3rd.	
	p-value	statistic	p-value	statistic	p-value	statistic	p-value	statistic	p-value	statistic
F1	1.0000	0.9498	1.0000	0.9144	1.0000	0.9494	1.0000	0.9720	1.0000	0.9286
Kappa	1.0000	0.9333	1.0000	0.8843	1.0000	0.9236	1.0000	0.9722	1.0000	0.8974
Specificity	1.0000	0.8559	1.0000	0.7408	1.0000	0.8497	1.0000	0.9018	1.0000	0.8145
Sensitivity	1.0000	0.9440	1.0000	0.8940	1.0000	0.9366	1.0000	0.9593	1.0000	0.9291
Precision	1.0000	0.9151	1.0000	0.8585	1.0000	0.9375	1.0000	0.9238	1.0000	0.9065

Supplementary Table 17: Performance indices of the classification framework (CNNCF) of the experiment P and the average performance of the 7th year respiratory resident (Respira.), the 3rd year emergency resident (Emerg.), the 1st year respiratory intern (Intern), the 5th year radiologist(Rad-5th) and the 3rd year radiologist(Rad-3rd).

	CT-data(*Normal and COVID-19 cases from LUNA and Youan Hospital)					
	CNNCF	Respire.	Emerg.	Intern.	Rad-5th	Rad-3rd
F1(95%CI)	1.0000<br>(1.0000,1.0000)	1.0000<br>(1.0000,1.0000)	1.0000<br>(1.0000,1.0000)	0.9048<br>(0.8000,0.9796)	1.0000<br>(1.0000,1.0000)	0.9500<br>(0.8649,1.0000)
Kappa(95%CI)	1.0000<br>(1.0000,1.0000)	1.0000<br>(1.0000,1.0000)	1.0000<br>(1.0000,1.0000)	0.8537<br>(0.7015,0.9655)	1.0000<br>(1.0000,1.0000)	0.9250<br>(0.8052,1.0000)
Specificity(95%CI)	1.0000<br>(1.0000,1.0000)	1.0000<br>(1.0000,1.0000)	1.0000<br>(1.0000,1.0000)	0.9250<br>(0.8333,1.0000)	1.0000<br>(1.0000,1.0000)	0.9750<br>(0.9117,1.0000)
Sensitivity(95%CI)	1.0000<br>(1.0000,1.0000)	1.0000<br>(1.0000,1.0000)	1.0000<br>(1.0000,1.0000)	0.9500<br>(0.8180,1.0000)	1.0000<br>(1.0000,1.0000)	0.9500<br>(0.8462,1.0000)
Precision(95%CI)	1.0000<br>(1.0000,1.0000)	1.0000<br>(1.0000,1.0000)	1.0000<br>(1.0000,1.0000)	0.8636<br>(0.7058,1.0000)	1.0000<br>(1.0000,1.0000)	0.9500<br>(0.8333,1.0000)
	CT-data(Pneumonia and COVID-19 cases from ICNP and Youan Hospital)					
	CNNCF	Respire.	Emerg.	Intern.	Rad-5th	Rad-3rd
F1(95%CI)	0.9756<br>(0.9129,1.0000)	1.0000<br>(1.0000,1.0000)	0.9048<br>(0.7856,0.9787)	0.8000<br>(0.6471,0.9091)	0.9744<br>(0.9143,1.0000)	0.7391<br>(0.5599,0.8627)
Kappa(95%CI)	0.9664<br>(0.8837,1.0000)	1.0000<br>(1.0000,1.0000)	0.8678<br>(0.7079,0.9690)	0.7158<br>(0.5356,0.8683)	0.9654<br>(0.8837,1.0000)	0.8229<br>(0.4069,0.7931)
Specificity(95%CI)	0.9818<br>(0.9375,1.0000)	1.0000<br>(1.0000,1.0000)	0.9455<br>(0.8793,1.0000))	0.8727<br>(0.7826,0.9584)	1.0000<br>(1.0000,1.0000)	0.8364<br>(0.7414,0.9259)
Sensitivity(95%CI)	1.0000<br>(1.0000,1.0000)	1.0000<br>(1.0000,1.0000)	0.9500<br>(0.8260,1.0000)	0.9000<br>(0.7500,1.0000)	0.9500<br>(0.8421,1.0000)	0.8500<br>(0.6667,1.0000)
Precision(95%CI)	0.9524<br>(0.8398,1.0000)	1.0000<br>(1.0000,1.0000)	0.8636<br>(0.6923,1.0000)	0.7200<br>(0.5263,0.8966)	1.0000<br>(1.0000,1.0000)	0.6538<br>(0.4583,0.8422)

Supplementary Table 18: Results of McNemar's Test for CNNCF and experts on distinct of COVID-19 and *Normal cases by means of CT-data collected from LUNA dataset and Youan Hospital

	CNNCF/Respira.		CNNCF/Emerg.		CNNCF/Intern.		CNNCF/Rad-5th.		CNNCF/Rad-3rd.	
	p-value	statistic	p-value	statistic	p-value	statistic	p-value	statistic	p-value	statistic
F1	1.0000	0.9000	1.0000	0.8502	1.0000	0.9268	1.0000	0.9474	1.0000	0.8571
Kappa	1.0000	0.9000	1.0000	0.8502	1.0000	0.9268	1.0000	0.9473	1.0000	0.8571
Specificity	1.0000	0.9000	1.0000	0.8502	1.0000	0.9268	1.0000	0.9048	1.0000	0.8571
Sensitivity	1.0000	0.9000	1.0000	0.8502	1.0000	0.9268	1.0000	0.9474	1.0000	0.8571
Precision	1.0000	0.7857	1.0000	0.7222	1.0000	0.8958	1.0000	0.9167	1.0000	0.6875

Supplementary Table 19: Results of McNemar's Test for CNNCF and experts on distinct of COVID-19 and Pneumonia cases by means of CT-data collected from ICNP dataset and Youan Hospital)

	CNNCF/Respira.		CNNCF/Emerg.		CNNCF/Intern.		CNNCF/Rad-5th.		CNNCF/Rad-3rd.	
	p-value	statistic	p-value	statistic	p-value	statistic	p-value	statistic	p-value	statistic
F1	1.0000	1.0000	1.0000	1.0000	1.0000	1.0000	1.0000	1.0000	1.0000	1.0000
Kappa	1.0000	1.0000	1.0000	1.0000	1.0000	1.0000	1.0000	1.0000	1.0000	1.0000
Specificity	1.0000	0.8484	1.0000	0.7911	1.0000	0.9318	1.0000	0.8750	1.0000	0.8235
Sensitivity	1.0000	1.0000	1.0000	1.0000	1.0000	1.0000	1.0000	1.0000	1.0000	1.0000
Precision	1.0000	0.9730	1.0000	0.9609	1.0000	1.0000	1.0000	0.9474	1.0000	1.0000

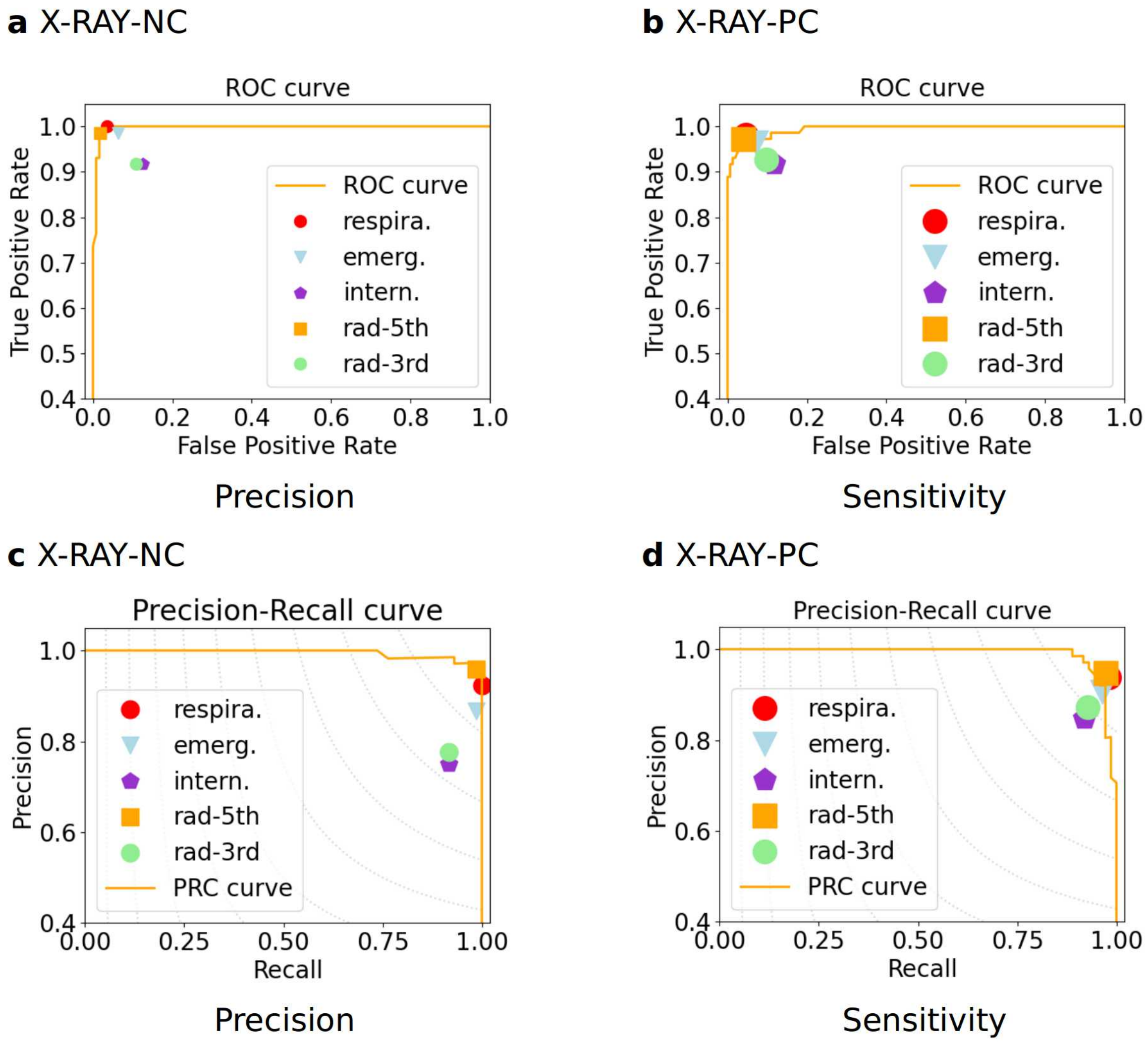

Supplementary Figure 12: ROC and PRC curves for the CNNCF and expert results for COVID-19 identification using XMVS. NC indicates that the positive case is a COVID-19 case, and the negative case is *Normal. PC indicates that the positive case is COVID-19, and the negative case is pneumonia. *Normal cases, pneumonia cases and COVID-19 cases used for evaluation were collected from both public data and Youan hospital data.

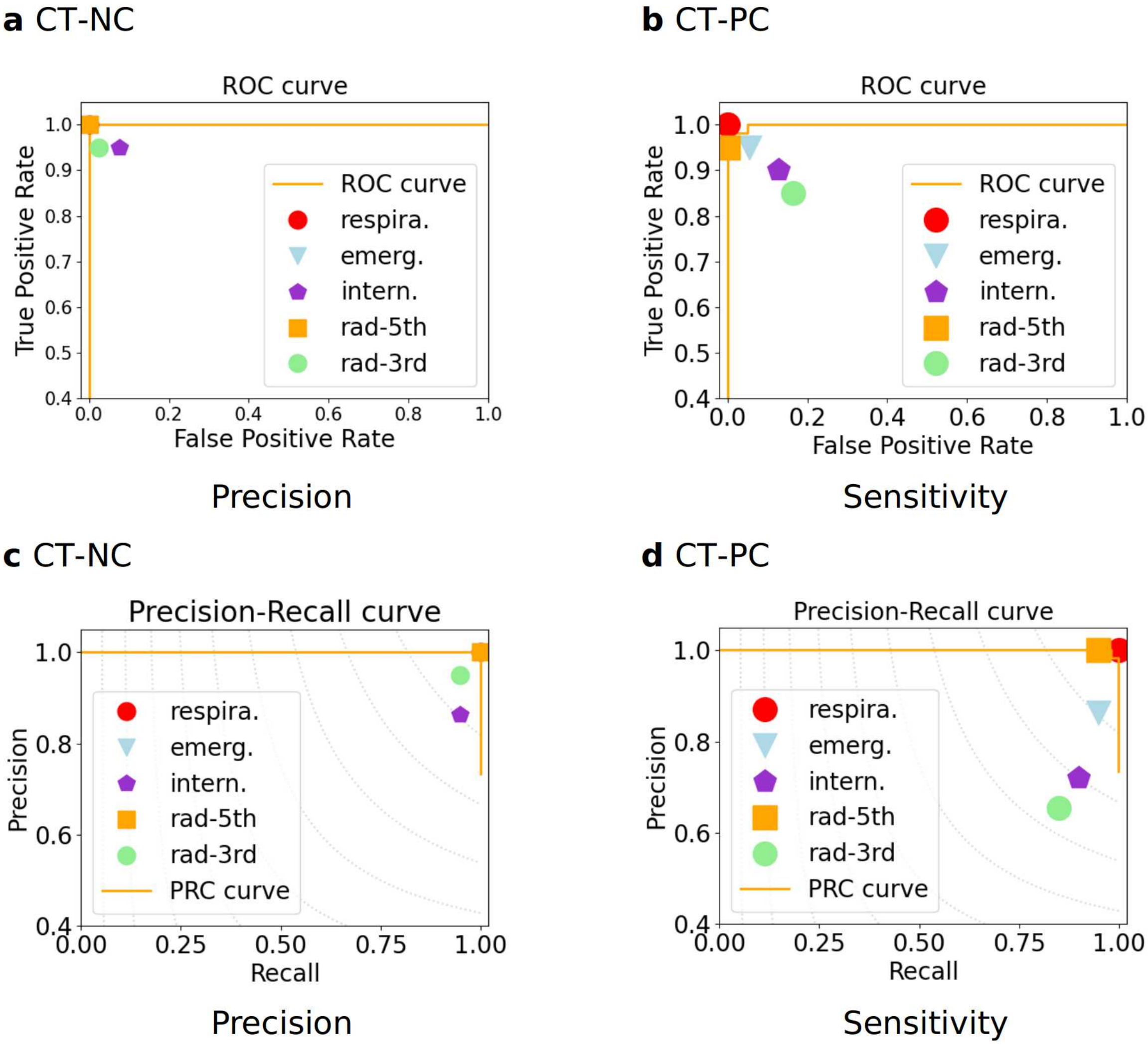

Supplementary Figure 13: ROC and PRC curves for the CNNCF and expert results for COVID-19 identification using CTMVS. NC indicates that the positive case is a COVID-19 case, and the negative case is *Normal. PC indicates that the positive case is COVID-19, and the negative case is pneumonia. *Normal cases and pneumonia cases used for evaluation were collected from both public data and Youan hospital data.

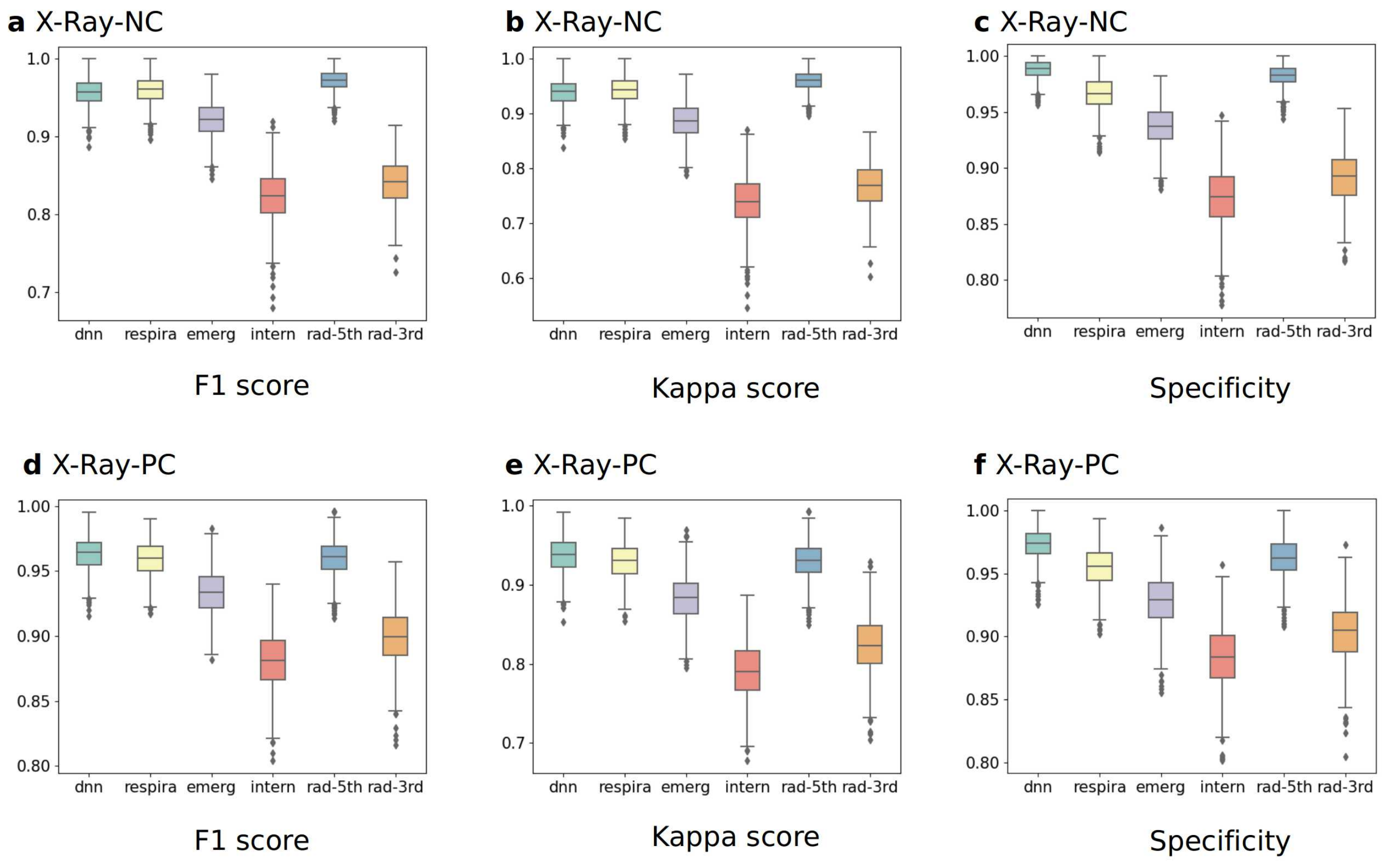

Supplementary Figure 14: Boxplots of F1 score, Kappa score and specificity for the CNNCF and expert results for COVID-19 identification on XMVS. NC indicates that the positive case is a COVID-19 case, and the negative case is *Normal. PC indicates that the positive case is COVID-19, and the negative case is Pneumonia. Bootstrapping is used to generate 1000 resampled validation sets for both XMVS and CTMVS.

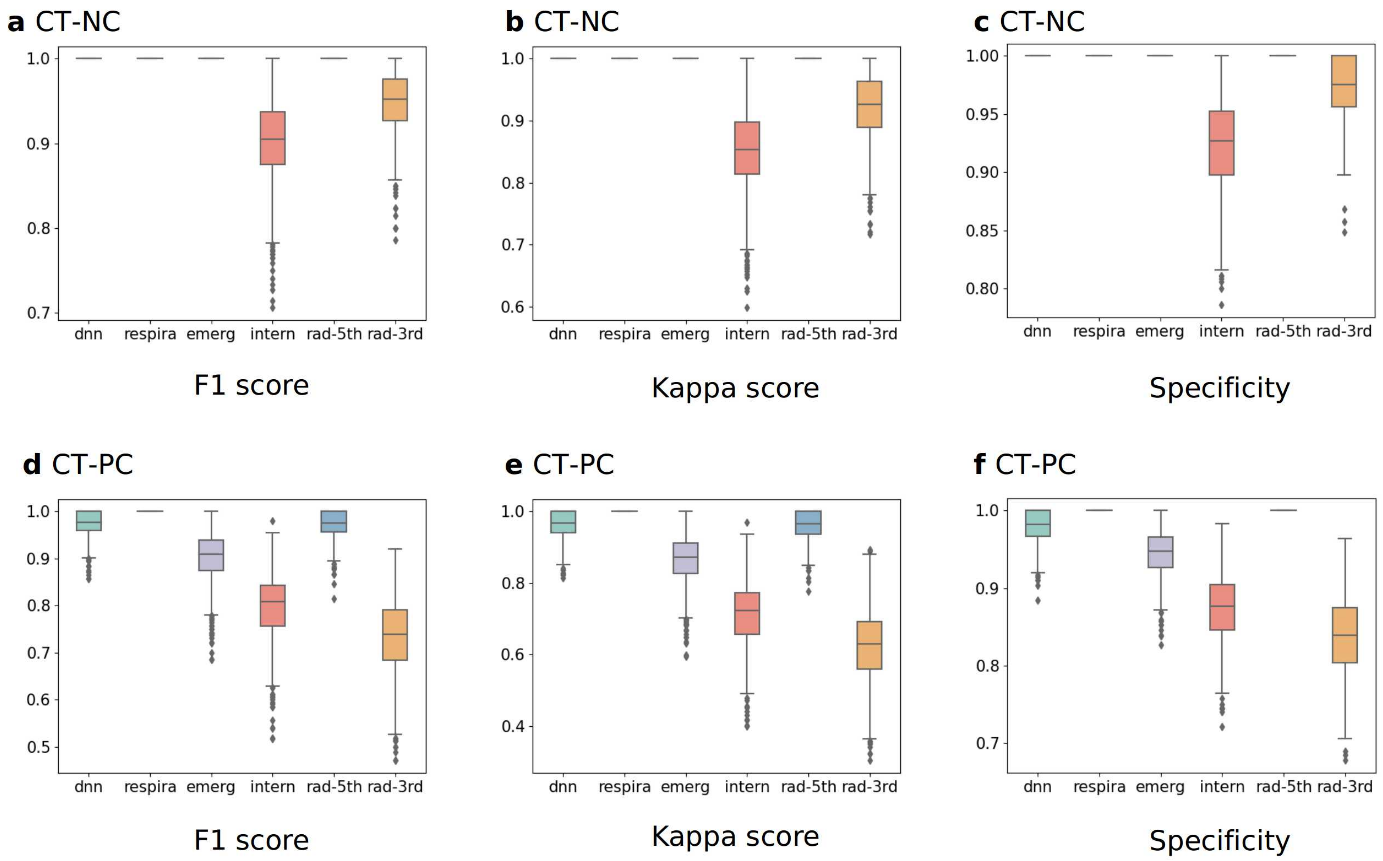

Supplementary Figure 15: Boxplots of F1 score, Kappa score and specificity for the CNNCF and expert results for COVID-19 identification on CTMVS. NC indicates that the positive case is a COVID-19 case, and the negative case is *Normal. PC indicates that the positive case is COVID-19, and the negative case is Pneumonia. Bootstrapping is used to generate 1000 resampled validation sets for both XMVS and CTMVS.

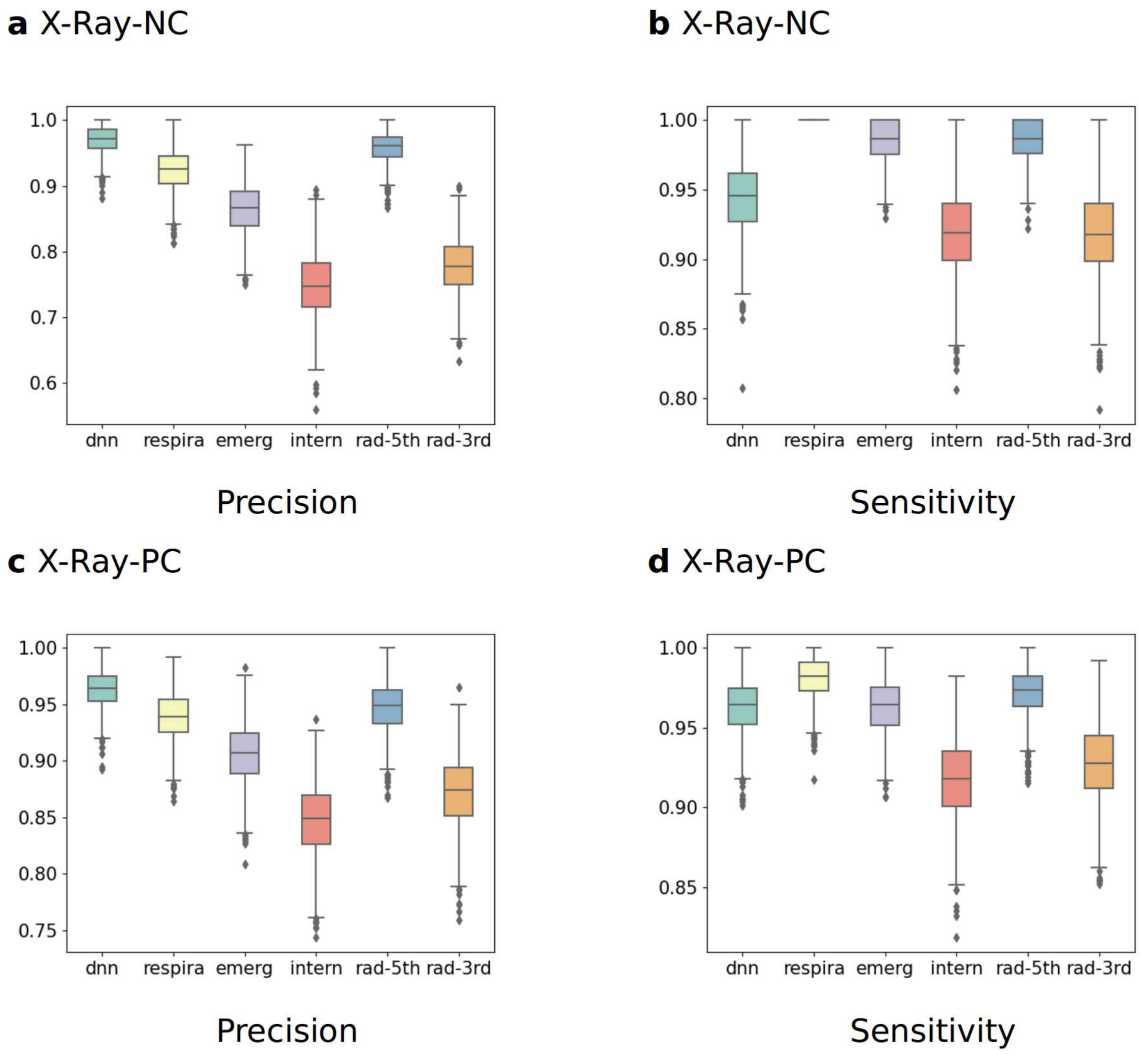

Supplementary Figure 16: Boxplots of precision and sensitivity for the CNNCF and expert results for COVID-19 identification on XMVS. NC indicates that the positive case is a COVID-19 case, and the negative case is *Normal. PC indicates that the positive case is COVID-19, and the negative case is Pneumonia. Bootstrapping is used to generate 1000 resampled validation sets for both XMVS and CTMVS.

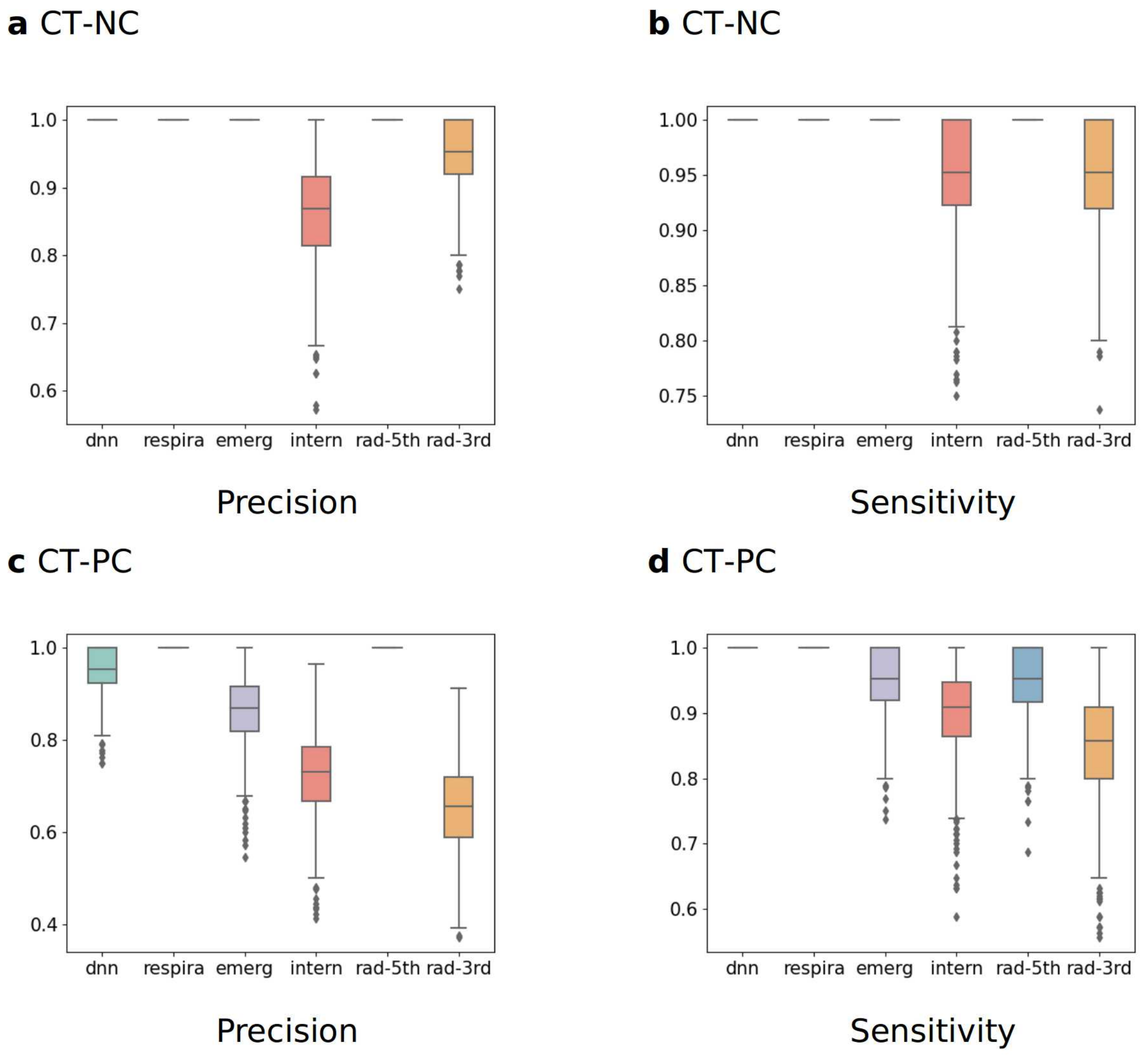

Supplementary Figure 17: Boxplots of precision and sensitivity for the CNNCF and expert results for COVID-19 identification on CTMVS. NC indicates that the positive case is a COVID-19 case, and the negative case is *Normal. PC indicates that the positive case is COVID-19, and the negative case is Pneumonia. Bootstrapping is used to generate 1000 resampled validation sets for both XMVS and CTMVS.

n. Experiment-R. In order to obatain a more comprehensive evaluation of the CNNCF while further improving the usability in clinical practice, the CNNCF was used to distinguish the COVID-19, pneumonia and *Normal cases simultaneously. The ROC scores for distinguishing COVID-19 from *Normal and pneumonia cases using XMVS are plotted in Supplementary Fig. 16-a; the AUROC of the CNNCF is 0.9714. The precision-recall scores for distinguishing COVID-19 from *Normal and pneumonia cases using X-data are shown in Supplementary Fig. 16-c; the AUPRC of the CNNCF is 0.9551. The ROC scores for distinguishing COVID-19 from *Normal and pneumonia cases using CTMVS are plotted in Supplementary Fig. 16-b; the AUROC of the CNNCF is 1.0. The precision-recall scores for distinguishing COVID-19 from *Normal and pneumonia cases are shown in Supplementary Fig. 16-d; the AUPRC of the CNNCF is 1.0.

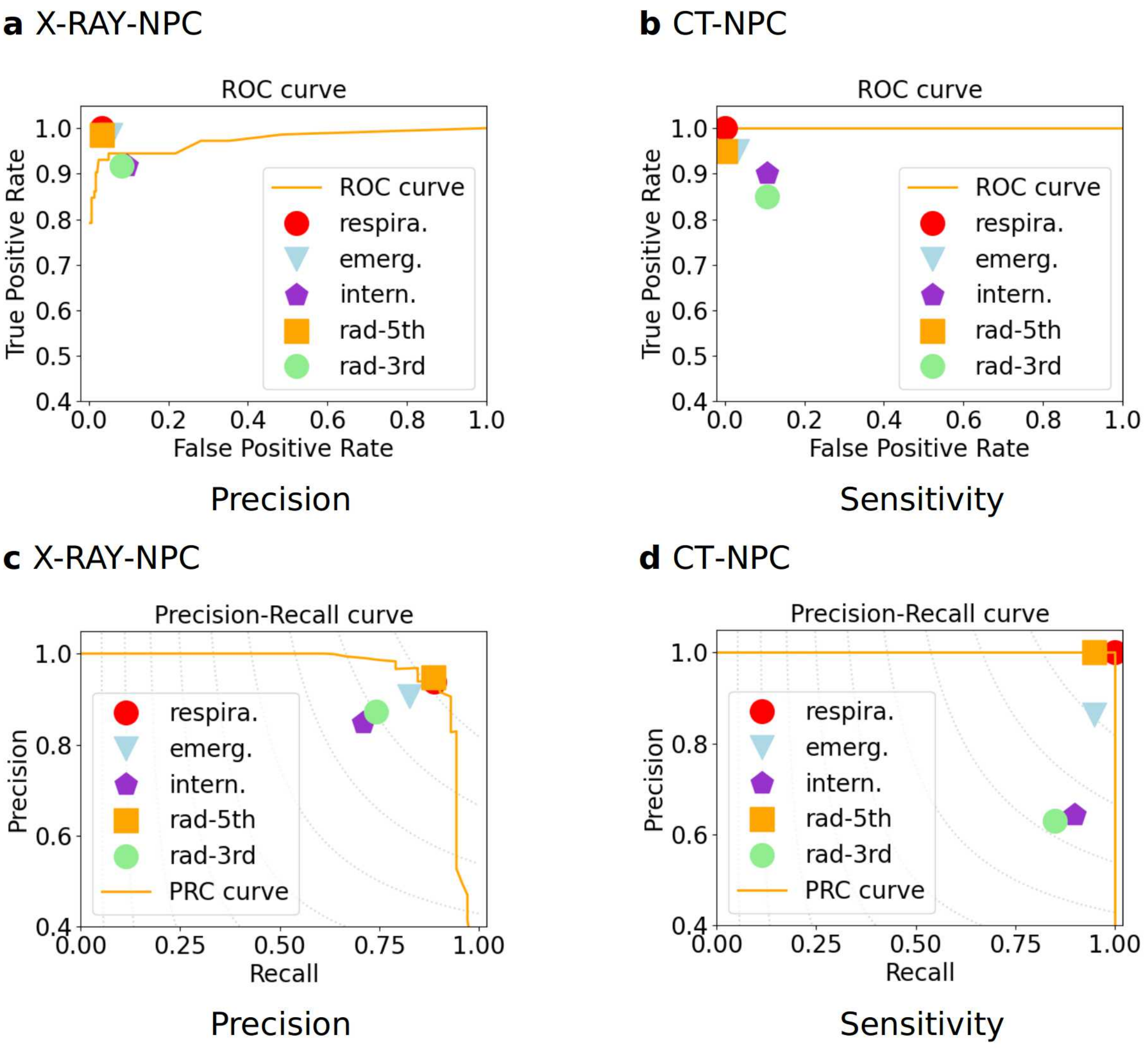

Supplementary Figure 18: ROC and PRC curves for the CNNCF and expert results for COVID-19 identification using XMVS and CTMVS. NPC indicates that the positive case is a COVID-19 case, and the negative case is *Normal and pneumonia. *Normal cases, pneumonia cases and COVID-19 cases used for evaluation were collected from both public data and Youan hospital data.

Supplementary Table 20: Five clinical indicators of COVID-19

Clinical indicators	COVID-19(n=95)
White blood cell (10^9/L)	4.26[3.50,5.82]
Neutrophil (%)	63.50[51.50,72.00]
Lymphocyte (%)	26.10[18.80,34.55]
Procalcitonin (mg/L)	0.12[0.10,0.15]
C-reactive protein (mg/L)	16.30[3.79,39.95]

Supplementary Methods

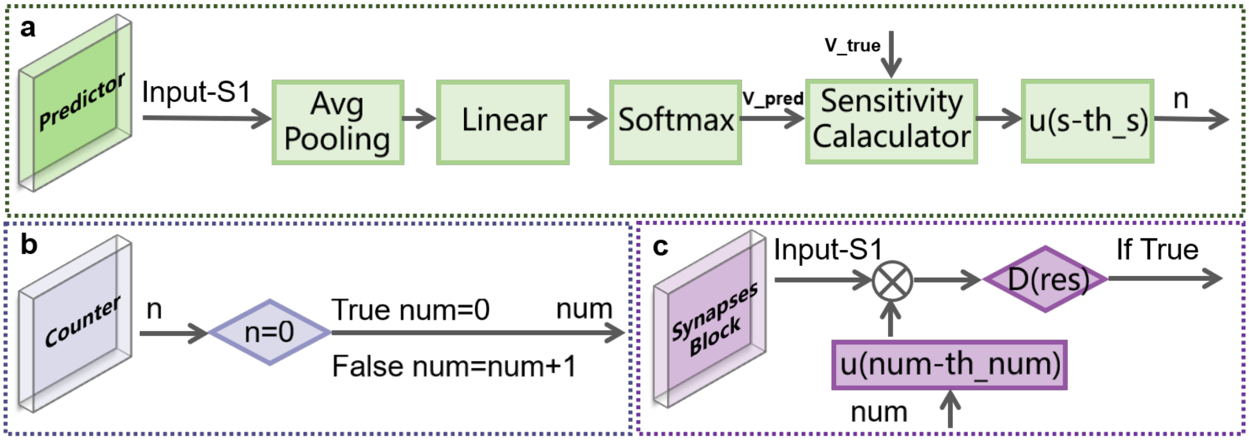

Supplementary Figure 19: Details of control Gate Block.

Supplementary Table 21: Hyper parameters of four teacher networks(TS)

			RT-PCR testing		
	ResBlock-A	ResBlock-B	Control Gate Block	ResBlock-A	ResBlock-B
TS1	2	1	1	3	1
TS2	2	2	1	2	1
TS3	3	1	1	2	1
TS4	3	2	1	2	1

Supplementary Table 22: Comparision of RT-PCR test results using throat specimen and the CNNCF results using CT data for COVID-19 and *Normal cases

	CNNCF	RT-PCR
F1(95%CI)	1.0000 (1.0000,1.0000)	0.9502 (0.9068,0.9790)
Kappa(95%CI)	1.0000 (1.0000,1.0000)	0.9229 (0.8574,0.9664)
Specificity(95%CI)	1.0000 (1.0000,1.0000)	1.0000 (1.0000,1.0000)
Sensitivity(95%CI)	1.0000 (1.0000,1.0000)	0.8947 (0.8295,0.9588)
Precision(95%CI)	1.0000 (1.0000,1.0000)	1.0000 (1.0000,1.0000)

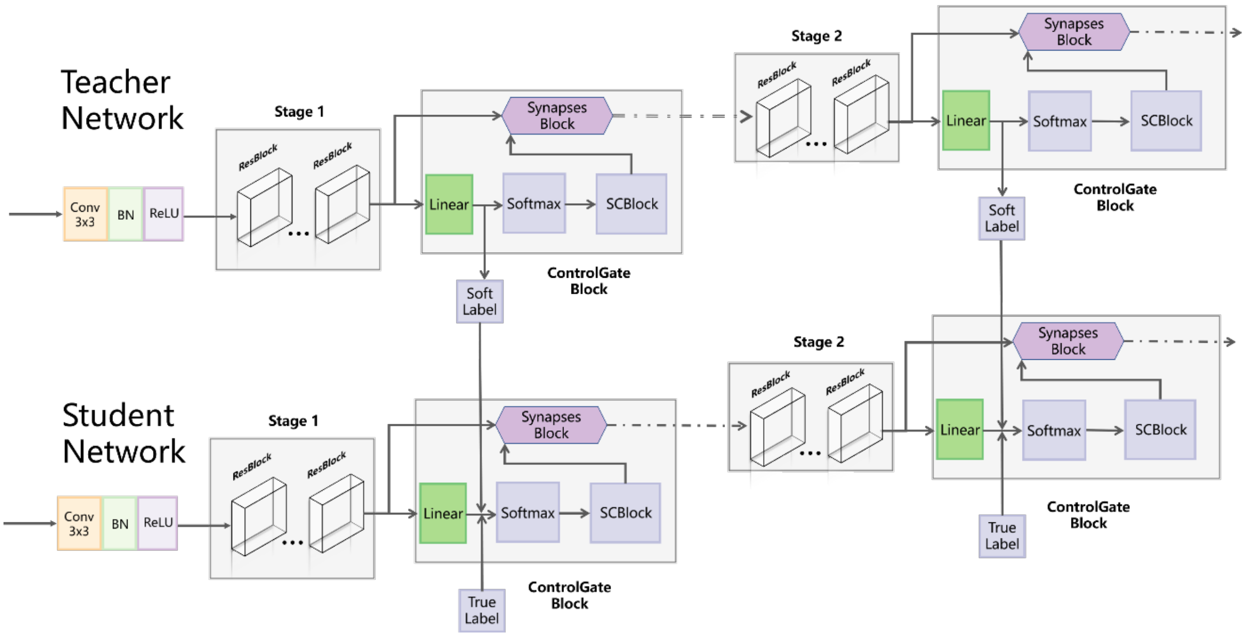

Supplementary Figure 20: Details of knowledge distilling method.

		**True condition**					
	Total population	Condition positive	Condition negative	Prevalence $= \frac{\sum \text{Condition positive}}{\sum \text{Total population}}$			
**Predicted condition**	Predicted condition positive	**True positive**, Power	**False positive**, Type | error	Positive predictive value (PPV), Precision $= \frac{\sum \text{True positive}}{\sum \text{Predicted condition positive}}$	False discovery rate (FDR) $= \frac{\sum \text{False positive}}{\sum \text{Predicted condition negative}}$		
	Predicted condition negative	**False negative**, Type || error	**True negative**	False omission rate (FOR) $= \frac{\sum \text{False negative}}{\sum \text{Predicted condition negative}}$	Negative predictive value (NPV) $= \frac{\sum \text{True negative}}{\sum \text{Predivted condtion negative}}$		
		True positive rate (TPR), Recall, Sensitivity, probability of detection $= \frac{\sum \text{True positive}}{\sum \text{Condition positive}}$	False positive rate (FPR), Fall-out, probability of false alarm $= \frac{\sum \text{False positive}}{\sum \text{Condition negative}}$	Positive likelihood ratio (LR+) $= \frac{\text{TPR}}{\text{FPR}}$	Diagnostic odds ratio (DOR) $= \frac{\text{LR+}}{\text{LR}-}$	F1 score $= \frac{2}{\frac{1}{\text{Recall}}+\frac{1}{\text{Precision}}}$	Accuracy (ACC) $= \frac{\sum \text{True positive}+\sum \text{True negative}}{\sum \text{Total population}}$
		False negative rate (FNR), Miss rate $= \frac{\sum \text{False negative}}{\sum \text{Condition positive}}$	True negative rate (TNR), Specificity (SPC) $= \frac{\sum \text{True negative}}{\sum \text{Condition negative}}$	Negative likelihood ratio (LR-) $= \frac{\text{FNR}}{\text{TNR}}$			

Supplementary Figure 21: The equations of the statistical indices.

Supplementary Abbreviations

Supplementary Table 23: Abbreviations

Abbreviations	Words and Phrases	Abbreviations	Words and Phrases
COVID-19	Coronavirus Disease 2019	CT	Computed Tomography
CNN	Convolutional Neural Network	WHO	World Health Organization
rRT-PCR	real-time Reverse TranscriptasePolymerase Chain Reaction	SOPs	Standard Operating Procedures
BSL-3	BioSafety Level 3	RNA	RiboNucleic Acid
ILI	Influenza-Like Illness	SARI	Severe Acute Respiratory Infection
CXR	Chest RadiogRaphy	DL	Deep Learning
SIFT	Scale-Invariant Feature Transform	RANSAC	Random Sample Consensus
PCA	Principal Component Analysis	Grad-CAM	Gradient-weighted Class Activation Mapping
TTSF	Train-Test-Split Function	DICOM	Digital Imaging and Communications in Medicine
OpenCV	Open Source Computer Vision Library	CNNCF	Convolutional Neural Network based Classification Framework
CNNRF	Convolutional Neural Network based Regression Framework	XPDS	X-ray Public DataSet
XPTS	X-ray Public Training Set	XPVS	X-ray Public Test Set
XHDS	X-ray Hospital DataSet	XHTS	X-ray Hospital Training Set
XHVS	X-ray Hospital Test Set	CTPDS	CT Public DataSet
CTPTS	CT Public Training Set	CTPVS	CT Public Test Set
CTHDS	CT Hospital DataSet	CTHTS	CT Hospital Training Set
CTHVS	CT Hospital Test Set	CADS	Correlation Analysis DataSet
CATS	Correlation Analysis Training Set	CAVS	Correlation Analysis Test Set
SAs	Suspected Areas with inflammatory lesions	XMTS	X-ray Mixed Training Set
XMVS	X-ray Mixed Test Set	CTMTS	CT Mixed Training Set
CTMVS	CT Mixed Test Set	CCD	COVID CXR Dataset
ROC	Receiver Operating Characteristic	AUROC	the Area Under the ROC curve
AUPRC	the Area Under the Precision-Recall Curve	DBSCAN	Density-Based Spatial Clustering of Applications with Noise
CPC	Center Pixel Coordinates	ST	Significance Test
MSE	Mean Square Error	MAE	Mean Absolute Error
RMSE	Root Mean Square Error	r	correlation coefficient
R^2	coefficient of determination	PCC	Pearson Correlation Coefficient
SGD	Stochastic Gradient Descent	JPG	Joint Photographic Experts Group
PNG	Portable Network Graphics	TIFF	Tag Image File Format
TPR	True Positive Rate	FPR	False Positive Rate
TP	True Positive	TN	True Negative
FN	False Negative	PPV	Positive Predictive Value
XNPDS	X-data of the *Normal cases in XPDS	XPPDS	X-data of the Pneumonia cases in XPDS
XCPDS	X-data of the COVID-19 cases in XPDS	XNHDS	X-data of the *Normal cases in XPHS
XPPDS	X-data of the Pneumonia cases in XPHS	XCPDS	X-data of the COVID-19 cases in XPHS
CTNPDS	CT-data of the *Normal cases in CTPDS	CTPPDS	CT-data of the Pneumonia cases in CTPDS
CTCPDS	CT-data of the COVID-19 cases in CTPDS	CTNHDS	CT-data of the *Normal cases in CTPHS
CTPPDS	CT-data of the Pneumonia cases in CTPHS	CTCPDS	CT-data of the COVID-19 cases in CTPHS
SQL	Structured Query Language	CSV	Comma-Separated Values
JSON	JavaScript object notation	Max-Pooling	Max-Pooling Layer
BN	batch norm layer	SRT	Standardized Residency Training
Kappa	Kappa score	Sen	Sensitivity
Spe	Specificity	Pr	Precision
Normal cases	cases where the lungs are not manifest evidence of COVID-19,	pneumonia or influenza on	imaging and the RT-PCR testing of the COVID-19 is negative.
COVID-19 cases	cases where the lungs are manifest evidence of COVID-19 on	imaging and the RT-PCR	testing of the COVID-19 is postive.
Influenza cases	cases where the lungs are manifest evidence of Influenza on	imaging and the RT-PCR	testing of the COVID-19 is negative.
Pneumonia cases	cases where the lungs are manifest evidence of Pneumonia on	imaging and the RT-PCR	testing of the COVID-19 is negative.

Supplementary References

[1] Zhu, J., Li, W. & Chen, L. Doctors in china: improving quality through modernisation of residency education. *The Lancet* **388**, 1922–1929 (2016).

[2] Huang, S.-L., Chen, Q. & Liu, Y. Medical resident training in china. *International Journal of Medical Education* **9**, 108 (2018).

[3] Zeng, M. S. *et al.* Current status of radiology in china. *World journal of gastroenterology* **6**, 193 (2000).
